# Improved Transition Management of Adolescents and Young Adults With Allergy and/or Asthma: An EAACI Task Force Report on a Follow‐Up European Survey

**DOI:** 10.1111/all.16603

**Published:** 2025-06-17

**Authors:** N. Atzert, C. Gore, R. C. Knibb, C. Alviani, E. Angier, K. Blumchen, P. Comberiati, B. Duca, A. DunnGalvin, T. Garriga‐Baraut, M. H. Gowland, B. Egmose, E. Khaleva, C. G. Mortz, H. Pite, O. Pfaar, M. Podesta, S. Sanchez Garcia, A. F. Santos, G. Roberts, M. Vazquez Ortiz

**Affiliations:** ^1^ Department of Allergy and Clinical Immunology Kinderspital Zurich Zurich Switzerland; ^2^ Department of Paediatric Allergy Imperial College Healthcare NHS Trust London UK; ^3^ Section of Inflammation, Repair and Development, National Heart and Lung Institute Imperial College London London UK; ^4^ Institute of Health and Neurodevelopment Aston University Birmingham UK; ^5^ Faculty of Medicine University of Southampton Southampton UK; ^6^ The David Hide Asthma and Allergy Research Centre St Mary's Hospital Isle of Wight UK; ^7^ Primary Care and Population Sciences University of Southampton Southampton UK; ^8^ Department of Pediatrics, Division of Pneumology, Allergology, Infectious Diseases and Gastroenterology Goethe University Frankfurt am Main Germany; ^9^ Department of Clinical and Experimental Medicine, Section of Paediatrics University of Pisa Pisa Italy; ^10^ Department of Child and Adolescent Health Mater Dei Hospital Msida Malta; ^11^ Applied Psychology and Paediatrics and Child Health University College Cork Cork Ireland; ^12^ Paediatrics and Child Infectious Diseases First Moscow State Medical University Moscow Russia; ^13^ Unitat d'Al·lergologia Pediàtrica Hospital Universitari Vall d'Hebron Barcelona Spain; ^14^ Grup d'Investigació “Creixement i Desenvolupament” Barcelona Spain; ^15^ Allergy Action St Albans UK; ^16^ Department of Dermatology and Allergy Centre, Odense Research Centre for Anaphylaxis (ORCA), Odense University Hospital University of Southern Denmark Odense Denmark; ^17^ Immuno‐Allergy Department CUF Tejo Hospital Lisbon Portugal; ^18^ NOVA Medical School Universidade NOVA de Lisboa Lisbon Portugal; ^19^ Department of Otorhinolaryngology, Head and Neck Surgery, Section of Rhinology and Allergy, University Hospital Marburg Philipps‐Universität Marburg Marburg Germany; ^20^ Food Allergy Italia APS Padova Italy; ^21^ European Federation of Allergy and Airways Diseases Patients' Associations (EFA) Brussels Belgium; ^22^ EAACI Patient Organisation Committee (POC) Zurich Switzerland; ^23^ Allergy Department Hospital Infantil Universitario del Niño Jesús Madrid Spain; ^24^ Department of Women and Children's Health (Paediatric Allergy), School of Life Course Sciences, Faculty of Life Sciences and Medicine King's College London London UK; ^25^ Peter Gorer Department of Immunobiology, School of Immunology and Microbial Sciences King's College London London UK; ^26^ Children's Allergy Service Guy's and St Thomas' Hospital London UK; ^27^ Asthma UK Centre in Allergic Mechanisms of Asthma London UK; ^28^ NIHR Southampton Biomedical Research Centre University Hospital Southampton NHS Foundation Trust Southampton UK; ^29^ David Hide Asthma and Allergy Centre Isle of Wight NHS Trust Newport UK

**Keywords:** adolescents, allergy, asthma, guidelines, teenager, transfer, transition, young adults, young people

## Abstract

**Background:**

Adolescents and young adults (AYA) with asthma and allergies have unexpectedly high mortality and morbidity. A survey in 2019 amongst healthcare professionals (HCPs) in Europe highlighted significant gaps in transition care, which negatively impacts patients' outcomes. Since then, an evidence‐based guideline and practical toolbox for effective transition of AYA with asthma and allergies have been published.

**Aims:**

To assess HCPs' perspectives, practice and challenges on transition care for AYA with asthma and allergies, including the impact of the recent guideline, potential differences across countries and changes since 2019.

**Methods:**

Cross‐sectional online survey‐based study. European HCP managing AYA with allergies and/or asthma were invited to participate in May–June 2023.

**Results:**

511 responses were collected. Amongst respondents, 60%–70% were familiar with the guideline and toolbox, and found them helpful. At least for selected patients, 50%–85% of respondents adhered to some guideline recommendations and initiatives/resources for transition care such as simplifying medication regimes, seeing AYA on their own or producing transition reports. We observed improvements compared to 2019 in areas such as prioritising transition, training, assessment of psychosocial issues and transition readiness, access to other HCP, transfer and post‐transfer feedback systems. However, 20% of respondents had no transition process and around 50% had no transition‐specific elements. Sixty percent found transition care ‘very’ or ‘moderately challenging’, with transition not being prioritised, time constraints and limited expertise on psychosocial issues as leading problems. Significant differences were observed in transition practice across countries.

**Conclusion:**

Despite improvement in training and approach towards transition care, challenges and limitations persist in effectively implementing evidence‐based guidelines. Raising greater awareness about the need for, and the positive impact of high‐quality transition care amongst policy‐makers, HCP, and patients/families remains a key priority to unlock resources for training and effective implementation at a national/international level.

## Graphical abstract

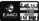

**Abbreviations**
AYA: adolescents and young adults
EAACI: European Academy of Allergy and Clinical Immunology
HCP: healthcare professionals
NAS: National Allergy Societies

## 1 Background

Asthma and allergic conditions, such as food allergies, eczema, allergic rhinoconjunctivitis and others, are significant health concerns amongst adolescents. The prevalence of food allergies in adolescents has been rising, with studies reporting rates between 4% and 7.1% over the past decade, up from 1% 20 years ago [1, 2, 3]. Supporting this trend, Venkataraman et al. found that food allergy prevalence in the UK increased from 2.3% at age 10 to 4% at age 18 [4]. Asthma rates in children aged 6–7 and 13–14 are around 10% with notable differences across countries and regions [5, 6].

Adolescence refers to a phase of development that typically begins around the ages of 10–11 and continues until the early 20s. It represents a transitional period with significant physical, social, emotional and cognitive changes, associated with transitions in education, social and other life aspects as well as ongoing maturation of the brain [7, 8].

Unexpectedly, this specific age group bears a substantial disease burden, including higher mortality rates resulting from allergic food reactions and inadequate asthma control [9, 10, 11]. Factors contributing to fatal outcomes include poor adherence to preventer medication in asthma [12], failing to correctly assess the severity of symptoms and delaying the administration of adrenaline in anaphylaxis [13]. Hence, health education is crucial for AYA to develop the necessary skills to manage their allergies and asthma appropriately as they become independent adults. Adolescence and early adulthood are critical periods for the onset or exacerbation of health‐risk behaviors and mental health issues, especially when combined with a chronic medical condition [14]. However, relatively little attention has been paid to AYA with chronic health problems compared to younger children and adults. AYAs are generally perceived as healthy and rarely seek medical attention.

Transition in healthcare refers to the planned shift of young individuals with long‐term conditions from a paediatric to an adult approach. Family, peers and healthcare professionals (HCPs) play a pivotal role during this process where the patient is empowered and supported to self‐manage their healthcare [12]. A survey amongst European HCPs conducted in 2019 by the EAACI Task Force on AYA showed significant deficiencies in transition management. A significant proportion lacked specialised training in this area. Moreover, the transition process often started late, after age 16–18, and a considerable number of HCPs expressed a lack of confidence in addressing psychosocial issues. Barriers to effective transition implementation included limited resources, lack of time, competing priorities and a misconception of transition as a mere transfer between paediatric and adult services. Only a minority of respondents had a transition programme in place, and many faced challenges in communication, availability of a transition lead and limited transition resources. Despite these difficulties, a significant proportion of respondents acknowledged that transition was important [15]. A recent publication from Sweden explored how young adults with severe asthma experienced the transition process. This highlighted the difficult balance between their perceived need to take responsibility and be involved in care, whilst feeling left out of the system and receiving insufficient support and engagement from HCP [16].

The EAACI Task Force on AYA with asthma and allergies published an evidence‐based guideline for effective transition for this patient population in 2020. Several dissemination initiatives were done, including an EAACI masterclass in 2022 and several dedicated symposia at international meetings. Key aspects of the guideline included initiating transition care early, around ages 11–13, using a structured multidisciplinary approach, providing comprehensive, accessible resources, as well as focusing on simplifying medication regimes, involving peers, addressing psychological and socio‐economic factors, encouraging family support, and promoting awareness within the broader community [17]. To facilitate the implementation of an effective transition, the Task Force published a practical Toolbox position paper in 2022 gathering pragmatic resources (mostly available in English) for HCP, AYA, families, schools, workplace and the wider community [18]. Additionally, expert narrative reviews on pragmatic approaches for transition in difficult asthma or food allergy have also been published [19, 20]. During the period from 2019 to 2023, the implementation of new guidelines in healthcare has been met with global threats such as the COVID‐19 pandemic. This crisis highlighted health disparities and social determinants of health, including income, education and healthcare access [21].

In this rapidly evolving landscape, the EAACI Task Force on AYA decided to conduct a new survey amongst HCP to assess the status of AYA transition in Europe and the UK. This survey aimed to analyse the current perspectives, training and practices related to transition care amongst HCPs across Europe, identify challenges that hinder the implementation of an effective transition strategy, assess differences across countries and any changes after the publication of the guideline and toolbox papers.

## 2 Methods

An observational cross‐sectional study using an online, anonymous survey for HCP was conducted. The survey was adapted from the former survey conducted by Khaleva et al. [15] and developed by the members of the EAACI AYA Task Force. New questions were included covering aspects related to awareness, perceived usefulness and implementation of the recommendations of the published guideline and Toolbox paper. Translation into nine European languages (English, German, French, Greek, Spanish, Portuguese, Turkish, Italian and Russian) was undertaken, including back translation to ensure validity, to facilitate dissemination and completion by HCPs.

We invited HCPs managing AYA with asthma and/or allergic conditions from EAACI and/or National Allergy Societies (NAS) in Europe. Inclusion criteria were HCPs managing AYA with allergy and/or asthma in their practice, and able to read any of the above‐mentioned languages. Respondents from regions outside Europe (i.e., not included within NAS or Russia) were excluded from analysis. Sample size was calculated with a known population size of 13.000 EAACI members. As an example of power, a sample size of 374 participants would provide a confidence interval of 7.1%–13.4% for a result of 10% (STATA v18).

The survey was distributed via a link using the Qualtrics platform. We followed a similar dissemination strategy to that successfully used in 2019 to minimize selection bias and obtain a representative sample of HCPs across Europe [15]. Dissemination was done by email to members via EAACI and National Allergy Societies, social media (Twitter, Facebook, LinkedIn) and conferences from 19th of May until 19th of June 2023. The study was approved by the Ethics and Research Governance Committee (ICREC Reference number: 6489853) at Imperial College London, United Kingdom. Quantitative variables were presented in a descriptive analysis as median and interquartile range (IQR) or mean and standard deviation (SD) based on normality test results. Categorical variables were presented as percentage and absolute numbers. A comparative analysis among the countries with over 30 responses in the survey was also performed to ensure that there was adequate power to detect significant differences.

Data obtained in 2023 versus 2019 was compared using parametric and/or nonparametric tests for independent groups as appropriate for the entire sample of respondents as well as for the countries with over 30 respondents in both surveys. Confounders were not adjusted in the above‐mentioned comparisons. PASW Statistics 27 (SPSS Inc.) was used. A two‐tailed nominal *p*‐value < 0.001 was considered statistically significant after applying Bonferroni correction for multiple comparisons.

## 3 Results

### 3.1 Respondent Characteristics and Demographics

We analysed 511 valid responses (Figure S1). Most respondents were doctors (80%) followed by specialist allergy nurses (10%). Most professionals worked in paediatric allergy (31%) or paediatrics (26%). Over 30% reported seeing patients of all age groups, and 12% identified themselves as adult physicians. The majority of participants were affiliated to the EAACI asthma (28%) or paediatrics (27%) sections and were mostly working in tertiary (47%) or secondary (37.4%) care. Respondents were mainly from the UK (31%, *n* = 157), France (15%, *n* = 77), Spain (13%, *n* = 67) and Germany (7%, *n* = 33) (Table 1).

**TABLE 1:** Respondent's characteristics and clinical settings.

Demographic and clinical setting characteristics of survey responders (%, *n*)	2023 (*n* = 511)	2019 (*n* = 1179)	*p*
EAACI section			< 0.001^b^
Asthma	28.2% (144)	24.8% (292)	
Dermatology	13.7% (70)	4.8% (57)	
ENT	5.3% (27)	3.9% (46)	
Immunology	7.3% (37)	8.4% (99)	
Paediatrics	27.6% (141)	30.4% (358)	
Primary care	6.3% (32)	4.3% (51)	
None	11.6% (59)	23.4% (276)	
Profession			< 0.001^b^
Doctor	79.6% (405)	91.8% (1082)	
Specialist allergy nurse	9.8% (50)	5.8% (68)	
Dietian	4.7% (24)	1.3% (15)	
Psychologist	3.9% (20)	0.3% (3)	
Other	2% (10)	0.9% (11)	
Specialty^a^			
Paediatric allergy	31.2% (159)	31.2% (368)	0.988^c^
Paediatrics	26.3% (134)	28.1% (331)	0.447^c^
Allergy (adults only)	11.8% (60)	11.7% (138)	0.972^c^
Allergy (children/adults)	30.8% (157)	43.6% (514)	< 0.001^c^
Dermatology	13.1% (67)	3.4% (40)	< 0.001^c^
Respiratory medicine	13.7% (70)	14.6% (172)	0.697^c^
ENT	5.9% (30)	3.1% (37)	0.008^c^
GP	7.5% (38)	3.5% (41)	< 0.001^c^
Gastroenterology	1.4% (7)	0% (0)	
Other	4.1% (21)	3% (35)	0.226^c^
Work setting^a^			
Tertiary care	46.5% (236)	46% (542)	0.854^c^
Secondary care	37.4% (190)	24.9% (293)	< 0.001^c^
Primary care	15.4% (78)	22.9% (270)	< 0.001^c^
Private practice	10.8% (55)	24% (283)	< 0.001^c^
Other	0.8% (4)	0.6% (7)	
Patient age group covered by HCP			0.003^b^
All ages	39.5% (202)	42.9% (506)	
0–14 years	6.5% (33)	2.6% (31)	
0–16 years	10% (51)	6.7% (79)	
0–18 years	24.1% (123)	28.8% (339)	
12 or over	4.1% (21)	2.9% (34)	
14 or over	2.3% (12)	2.5% (30)	
16 or over	3.9% (20)	4.3% (51)	
18 or over	7% (36)	7.5% (88)	
Other	2.5% (13)	1.6% (19)	
Time for follow‐up consultation			0.104^b^
Up to 10 min	12% (61)	11.5% (135)	
Up to 20 min	34.8% (177)	39% (460)	
Up to 30 min	35.6% (181)	33.5% (395)	
Up to 45 min	11% (56)	12.1% (143)	
Over 45 min	6.5% (33)	3.9% (46)	
Direct access to other HCPs^a^			
Allergy/asthma nurse	54.9% (256)	50.6% (597)	0.12^c^
Dietician	43.6% (203)	32.1% (379)	< 0.001^c^
Paediatric allergist	55.4% (258)	45.5% (537)	< 0.001^c^
Adult allergist	42.5% (198)	37.1% (437)	0.042^c^
Psychologist	35.4% (165)	24.9% (293)	< 0.001^c^
Respiratory physio	30% (140)	23.7% (279)	0.007^c^
Social worker	27.9% (130)	17.7% (209)	< 0.001^c^
Gastroenterology	31.1% (145)	36.1% (426)	0.054^c^
Pulmonologist	34.3% (160)	46.1% (543)	< 0.001^c^
Dermatologist	42.3% (197)	42.6% (502)	0.911^c^
Audiologist	24.7% (115)	27.9% (329)	0.184^c^
Ophthalmologist	27.7% (129)	0.0% (0)	
Specific training in AYA care^a^			
Dedicated programme	28.3% (142)	11.7% (138)	< 0.001^c^
Short training	40.9% (205)	4.1% (48)	< 0.001^c^
Supervison within clinic	35.1% (176)	13.7% (161)	< 0.001^c^
No training	41.5% (208)	75.7% (892)	< 0.001^c^
Age when to start transition			< 0.001^b^
10–12 years	7.4% (34/458)	2% (23/1154)	
12–14 years	11.8% (54/458)	9.5% (110/1154)	
14–16 years	14% (64/458)	17.5% (202/1154)	
16–18 years	14.8% (68/458)	27.6% (318/1154)	
Depends on patient	30.6% (140/458)	1.9% (22/1154)	
Other	1.1% (5/458)	0.3% (3/1154)	
Transition is important			< 0.000^b^
Strongly agree	42.8% (217/507)	54.5% (621/1139)	
Agree	42.2% (214/507)	32.2% (367/1139)	
Neither agree nor disagree	12.4% (63/507)	10.9% (124/1139)	
Disagree	2% (10/507)	1.8% (21/1139)	
Strongly disagree	0.6% (3/507)	0.5% (6/1139)	
Transition in country/priority			< 0.001^b^
Yes	43.8% (221/504)	16.7% (197/1179)	
No	37.1% (187/504)	40.3% (475/1179)	
Do not know	19% (96/504)	43% (507/1179)	

Demographic and clinical setting characteristics comparison by four main countries in 2023 survey (%, *n*)					
	UK	France	Spain	Germany	*p*
	*n* = 157	*n* = 77	*n* = 67	*n* = 33	
EAACI section					< 0.001^d^
Asthma	28.8% (45)	44.2% (34)	16.4% (11)	36.4% (12)	
Dermatology	14.1% (22)	19.5% (15)	4.5% (3)	18.2% (6)	
ENT	3.2% (5)	5.2% (4)	0% (0)	15.2% (5)	
Immunology	10.9% (17)	2.6% (2)	0% (0)	3% (1)	
Paediatrics	26.3% (41)	11.7% (9)	44.8% (30)	24.2% (8)	
Primary care	4.5% (7)	16.9% (13)	1.5% (1)	3% (1)	
None	12.2% (19)	0% (0)	32.8% (22)	0% (0)	
Profession					< 0.001^d^
Doctor	72.9% (113)	83.1% (64)	97% (65)	90.9% (30)	
Specialist allergy nurse	12.3% (19)	7.8% (6)	3% (2)	0% (0)	
Dietian	4.5% (7)	0% (0)	0% (0)	3% (1)	
Psychologist	4.5% (7)	9.1% (7)	0% (0)	6.1% (2)	
Other	5.8% (9)	0% (0)	0% (0)	0% (0)	
Specialty^a^					
Paediatric allergy	39.1% (61)	3.9% (3)	56.7% (38)	30.3% (10)	< 0.000^d^
Paediatrics	34% (53)	10.4% (8)	41.8% (28)	24.2% (8)	< 0.000^d^
Allergy (adults only)	10.3% (16)	9.1% (7)	3% (2)	15.2% (5)	0.189^d^
Allergy (children/adults)	17.9% (28)	44.2% (34)	35.8% (24)	30.3% (10)	< 0.001^d^
Dermatology	18.6% (29)	24.7% (19)	3% (2)	18.2% (6)	0.005^d^
Respiratory medicine	17.9% (28)	9.1% (7)	6% (4)	30.3% (10)	0.003^d^
ENT	5.1% (8)	10.4% (8)	0% (0)	18.2% (6)	0.003^d^
GP	9.6% (15)	7.8% (6)	0% (0)	3% (1)	0.048^d^
Gastroenterology	1.3% (2)	2.6% (2)	0% (0)	0% (0)	0.477^d^
Other	6.4% (10)	2.6% (2)	1.5% (1)	0% (0)	0.148^d^
Work setting^a^					
Tertiary care	45.2% (70)	51.9% (40)	49.3% (33)	51.5% (17)	0.757^d^
Secondary care	50.3% (78)	37.7% (29)	37.3% (25)	12.1% (4)	0.001^d^
Primary care	12.9% (20)	10.4% (8)	4.5% (3)	24.2% (8)	0.033^d^
Private practice	3.2% (5)	1.3% (1)	16.4% (11)	15.2% (5)	< 0.000
Other	1.9% (3)	0% (0)	0% (0)	0% (0)	0.326^d^
Patient age group covered by HCP					
All ages	35% (55)	77.9% (60)	37.3% (25)	30.3% (10)	
0–14 years	1.9% (3)	3.9% (3)	26.9% (18)	9.1% (3)	
0–16 years	17.8% (28)	0% (0)	20.9% (14)	0% (0)	
0–18 years	24.8% (39)	16.9% (13)	10.4% (7)	27.3% (9)	
12 or over	8.3% (13)	0% (0)	0% (0)	6.1% (2)	
14 or over	1.3% (2)	0% (0)	3% (2)	6.1% (2)	
16 or over	5.7% (9)	0% (0)	0% (0)	6.1% (2)	
18 or over	3.2% (5)	1.3% (1)	1.5% (1)	6.1% (2)	
Other	1.9% (3)	0% (0)	0% (0)	9.1% (3)	
Time for follow‐up consultation					< 0.001^b^
Up to 10 min	12.2% (19)	5.2% (4)	23.9% (16)	12.5% (4)	
Up to 20 min	38.5% (60)	9.1% (7)	53.7% (36)	56.3% (18)	
Up to 30 min	37.2% (58)	41.6% (32)	16.4% (11)	28.1% (9)	
Up to 45 min	9% (14)	22.1% (17)	1.5% (1)	3.1% (1)	
Over 45 min	3.2% (5)	22.1% (17)	4.5% (3)	0% (0)	
Direct access to other HCPs^a^					
Allergy/asthma nurse	52.1% (75)	71.4% (55)	74.1% (43)	43.3% (13)	0.001^d^
Dietician	40.3% (58)	71.4% (55)	17.2% (10)	53.3% (16)	< 0.001^d^
Paediatric allergist	45.1% (65)	72.7% (56)	67.2% (39)	46.7% (14)	< 0.001^d^
Adult allergist	32.6% (47)	62.3% (48)	44.8% (26)	53.3% (16)	< 0.001^d^
Psychologist	32.6% (47)	70.1% (54)	25.9% (15)	36.7% (11)	< 0.001^d^
Respiratory physio	25% (36)	61% (47)	20.7% (12)	36.7% (11)	< 0.001^d^
Social worker	24.3% (35)	64.9% (50)	19% (11)	23.3% (7)	< 0.001^d^
Gastroenterology	13.2% (19)	63.6% (49)	46.6% (27)	30% (9)	< 0.001^d^
Pulmonologist	13.2% (19)	62.3% (48)	55.2% (32)	46.7% (14)	< 0.001^d^
Dermatologist	29.9% (43)	70.1% (54)	50% (29)	46.7% (14)	< 0.001^d^
Audiologist	8.3% (12)	61% (47)	20.7% (12)	36.7% (11)	< 0.001^d^
Ophthalmologist	11.1% (16)	58.4% (45)	41.4% (24)	26.7% (8)	< 0.001^d^
Specific training in AYA care^a^					
Dedicated programme	31.8% (49)	72.7% (56)	7.5% (5)	24.2% (8)	< 0.001^d^
Short training	59.7% (92)	76.6% (59)	13.4% (9)	18.2% (6)	< 0.001^d^
Supervison within clinic	39.6% (61)	72.7% (56)	10.4% (7)	18.2% (6)	< 0.001^d^
No training	24.7% (38)	18.2% (14)	82.1% (55)	57.6% (19)	< 0.001^d^
Age when to start transition					< 0.001^b^
No transition process	15.6% (23)	1.4% (1)	31.1% (19)	24.1% (7)	
10–12 years	6.8% (10)	8.2% (6)	4.9% (3)	17.2% (5)	
12–14 years	17.7% (26)	8.2% (6)	18% (11)	10.3% (3)	
14–16 years	12.9% (19)	2.7% (2)	24.6% (15)	17.2% (5)	
16–18 years	8.8% (13)	9.6% (7)	11.5% (7)	13.8% (4)	
Depends on patient	35.4% (52)	69.9% (51)	8.2% (5)	17.2% (5)	
Other	2.7% (4)	0% (0)	1.6% (1)	0% (0)	
Transition is important					< 0.001^b^
Strongly agree	40.8% (64)	20.8% (16)	68.7% (46)	51.5% (17)	
Agree	37.6% (59)	67.5% (52)	26.9% (18)	36.4% (12)	
Neither agree nor disagree	17.8% (28)	10.4% (8)	3% (2)	9.1% (3)	
Disagree	3.2% (5)	1.3% (1)	1.5% (1)	0% (0)	
Strongly disagree	0.6% (1)	0% (0)	0% (0)	3% (1)	
Transition in country/priority					< 0.001^b^
Yes	49.7% (78)	88.3% (68)	11.9% (8)	24.2% (8)	
No	31.8% (50)	6.5% (5)	67.2% (45)	54.5% (18)	
Do not know	18.5% (29)	5.2% (4)	20.9% (14)	21.2% (7)	

*Note:* Limited responses were obtained from Latvia, Finland (*n* = 1); Estonia and Greece (*n* = 2); Cyprus, Slovenia and Sweden (*n* = 3); Czech Republic (*n* = 4); Turkey, Ireland and Slovakia (*n* = 5); more responses were obtained from: Romania (*n* = 6); Netherlands, Portugal, and Ukraine (*n* = 7); Belarus and Denmark (*n* = 8); Bulgaria and Croatia (*n* = 9); Belgium (*n* = 14); Russia (*n* = 16); Austria, Italy and Switzerland (*n* = 17); Germany (*n* = 33); Spain (*n* = 67); France (*n* = 77); UK (*n* = 157); Other European‐countries: (*n* = 0).

Abbreviations: ENT, otolaryngology; GP, general practitioner.

^a^ Participants were allowed to select more than one answer.

^b^ Chi‐squared.

^c^ Mann–Whitney *U*.

^d^ Kruskall–Wallis.

Transition was reportedly seen as a priority in their country by 44% of respondents, (see Table 1). Most respondents agreed (42%) or strongly agreed (43%) with the statement ‘transition is important’. Seventy‐nine percent of respondents indicated that they had a transition process in their practice. Fifty‐nine percent of respondents reported some training in transition care. Forty‐one percent had completed short training, 35% supervision in clinic and 28% a dedicated programme (Table 1).

### 3.2 Familiarity and Adoption of EAACI Guidelines and Transition Practice

**FIGURE 1:**
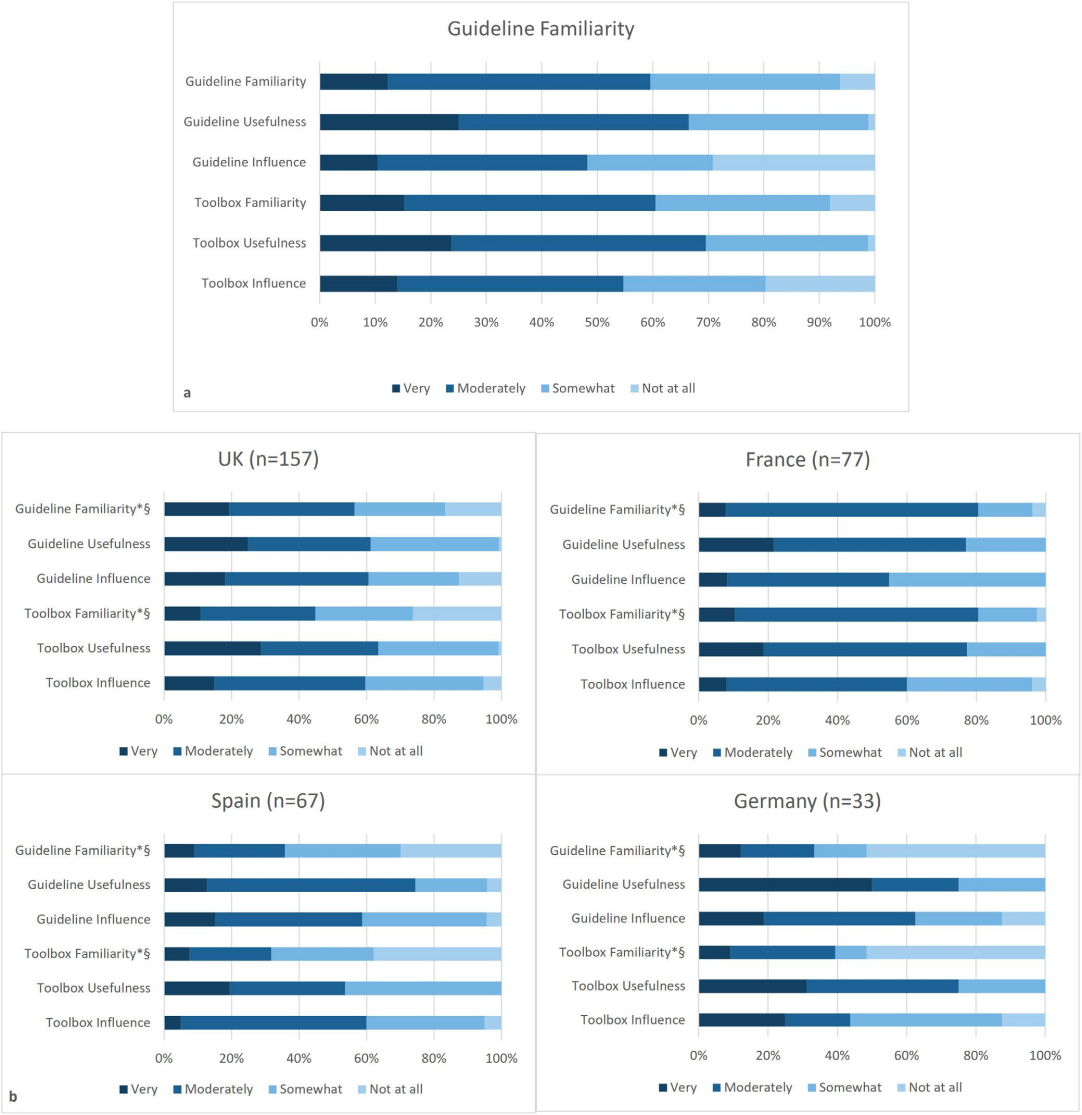
Familiarity, usefulness and influence in clinical practice of EAACI Guidelines and Practical Toolbox amongst HCP on the effective transition of adolescents for (a) all respondents and (b) separately for countries with over 30 responses in the survey. HCP, healthcare professionals; 511 participants contributed to the statistical analysis. UK: *n* = 157, France: *n* = 77, Spain: *n* = 67, Germany: *n* = 33 respondents. Symbols: *p value < 0.001; ^§^Kruskall–Wallis test (variables converted into quantitative scale). For comparison across countries for all other items *p* value was > 0.05.

Awareness and perceived usefulness of EAACI guidelines and toolbox. Sixty to seventy percent of respondents reported being ‘very’ or ‘moderately familiar’ with the guideline and toolbox and finding them ‘very’ or ‘moderately helpful’. Around 50% reported the guideline and toolbox had influenced their practice (Figure 1a).

#### 3.2.1 Adherence to EAACI Guideline Recommendations

**FIGURE 2:**
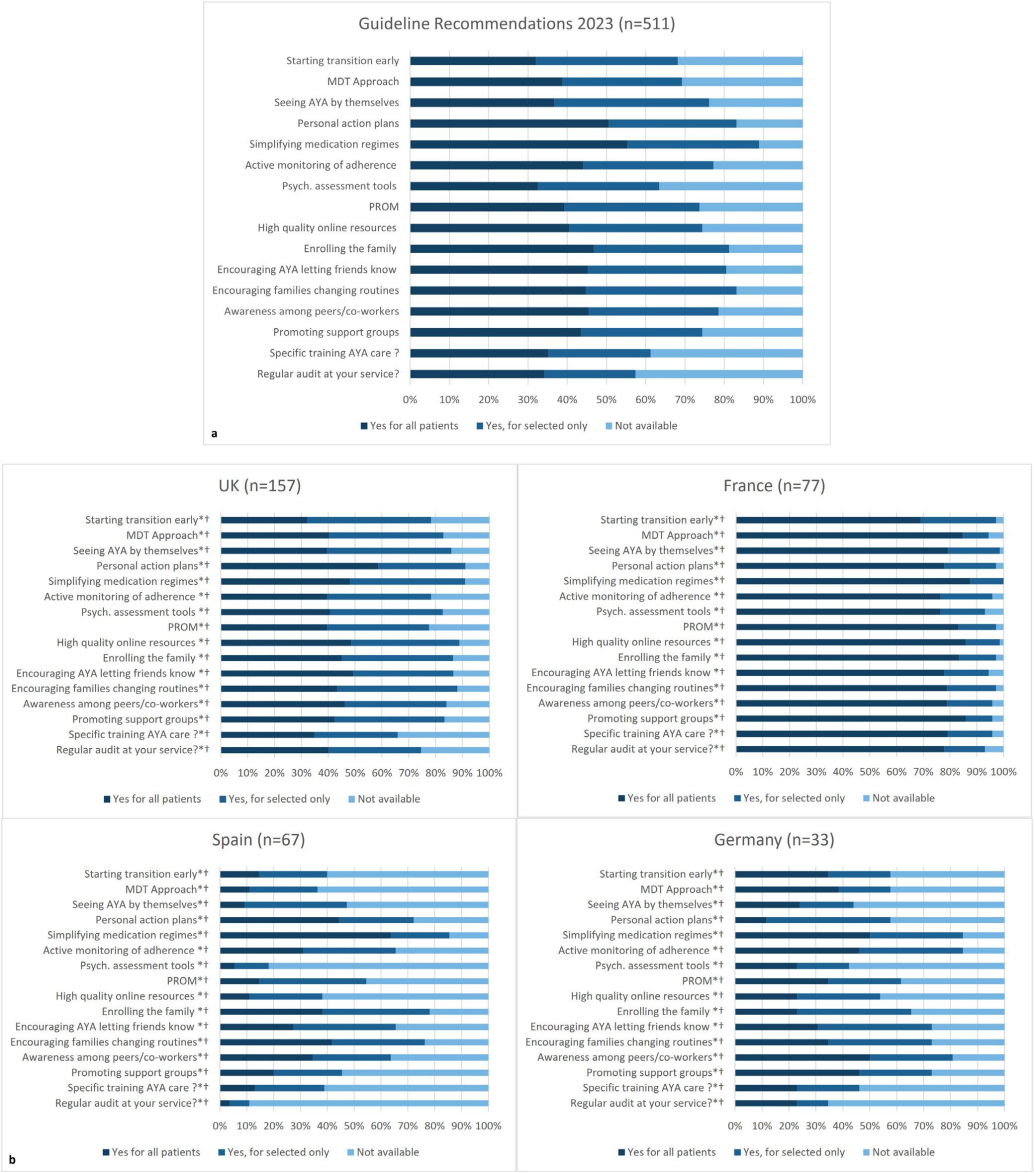
Available transition components according to EAACI Guideline recommendations used by HCP in clinical practice for (a) all respondents and (b) countries with over 30 responses in the survey. Abbreviations: AYA, adolescents and young adults; MDT, Multidisciplinary approach; PROM, patient‐reported outcome measure. 511 participants contributed to the statistical analysis. UK: *n* = 157, France: *n* = 77, Spain: *n* = 67, Germany: *n* = 33 respondents. Symbols: *p value < 0.001; ^†^Chi‐squared test.

Many respondents reported adopting strategies in line with the 2020 EAACI guideline recommendations for ‘all’ (32%–55%) or ‘selected patients’ (23%–39%) (Figure 2a). The recommendations most adopted included simplifying medication regimes (reported by 89% of respondents), encouraging change in family routines to promote adherence (83%), issuing personal action plans (83%) and enrolling the family in the transition process (81%). A proportion of respondents (11%–42%) reported such strategies not being available or implemented in their practice. For instance, regular audits of transition care/service were not available for 42% of respondents, specific training in AYA care for 38%, psychosocial assessment tools for 36% or starting transition early for 31%. Thirty percent indicated that the process starting age is individualised and only 19% started at around age 10–14 years.

**FIGURE 3:**
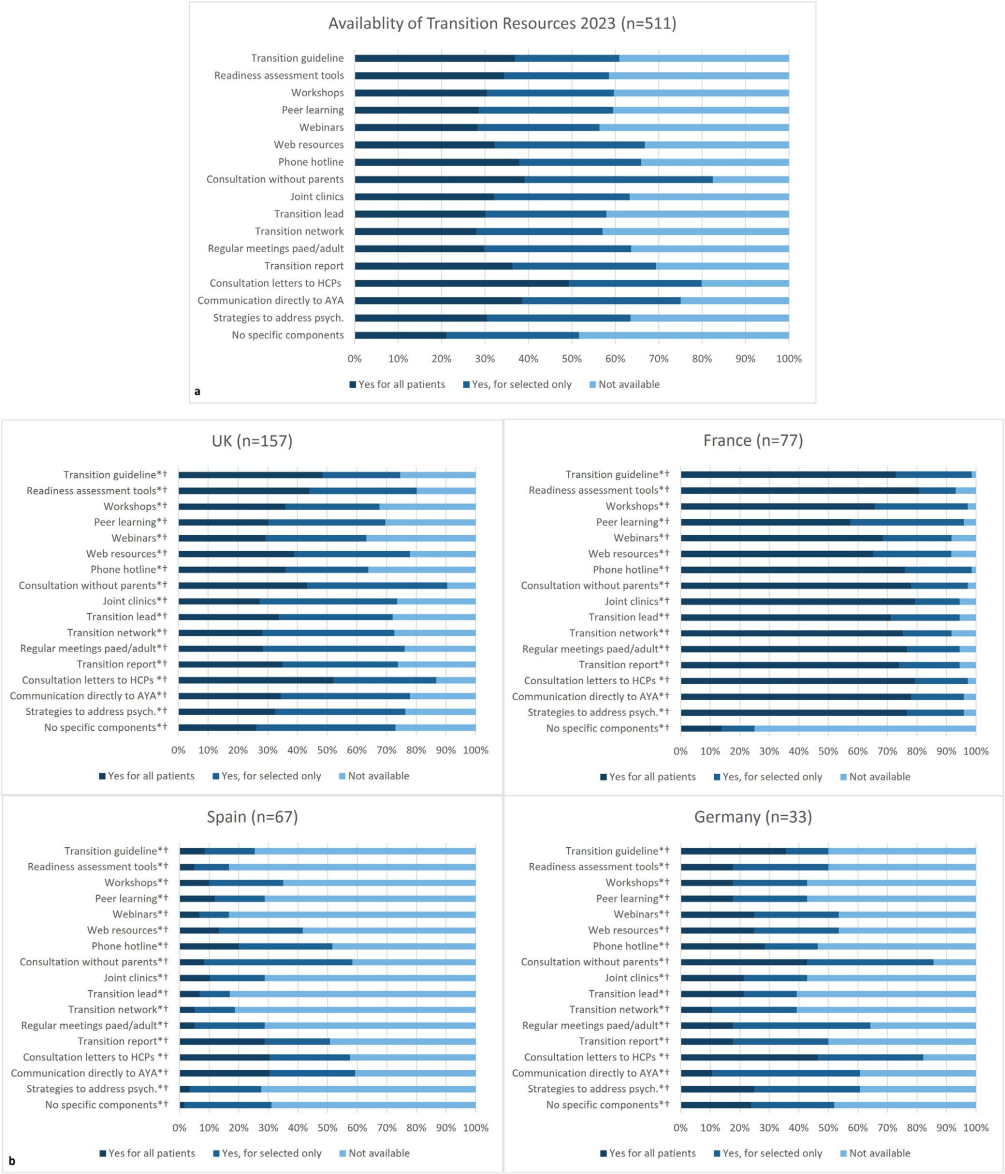
Availability of transition resources for HCPs in their service for (a) all respondents and (b) amongst countries with over 30 responses in the survey. UK: *n* = 157, France: *n* = 77, Spain: *n* = 67, Germany: *n* = 33 respondents. Abbreviations: AYA, adolescents and young adults; HCP, healthcare professionals. 511 participants contributed to the statistical analysis. Symbols: *p value < 0.001; ^†^Chi‐squared test.

Availability of transition initiatives and resources for HCPs. Many respondents reported having dedicated transition resources in their service for ‘all’ (21%–49%) or ‘selected patients’ (24%–43%) (Figure 3a). Most reported seeing AYA on their own (83%). Most generated consultation letters for HCP (80%) and transition reports (69%). In contrast, a transition guideline, transition readiness assessment tools, a transition network and/or transition lead was not available for around 40% of respondents.

Identifying and addressing psychosocial issues. Over 70% of respondents asked their AYA patients ‘always’ or ‘often’ about adherence, self‐management, smoking and family. Over 70%–80% felt ‘very’ or ‘quite confident’ when asking and/or giving advice about those aspects, as well as about sleep, alcohol, hobbies and education. However, over 50% reported asking only ‘sometimes’ or ‘never’ about depression, bullying, self‐harm, relationships or sexuality. Over 30%–50% reported feeling ‘not very confident’ or ‘not confident’ when asking and/or giving advice about those aspects or about psychological issues (see Figure S2a). Access to other HCP is summarised in Table 1.

Transfer from paediatric to adult service. Most respondents (around 60%) reported transferring 10%–50% of their patients to adult services, whilst 26% reported not transferring patients due to seeing all ages in their practice (Table 2). The transfer age was highly variable, with 24% of respondents indicating this was an individualised decision. The most common criteria/scenarios for transfer (reported by 45%–56% of respondents) included multiple food allergy and asthma with/without adrenaline autoinjector; difficult or severe asthma; biological treatment for asthma or chronic spontaneous urticaria/angioedema; uncontrolled atopic dermatitis and eosinophilic oesophagitis. Regarding readiness assessment prior to transfer, 34% of respondents reported using a checklist of questions or transition tool (significantly higher than the 4% reported in 2019, *p* < 0.001) and 22% had a transition committee. Fifty‐nine percent reported some form of feedback mechanism following transfer. Forty‐four percent reported regular meetings and 40% received consultation letters from the adult services. Strategies to foster continuity of care throughout transition and transfer were reported by 30%–58% of respondents, with transition reports, action plans and notification of transfer to primary care being the most common initiatives (Table 2).

**TABLE 2:** Characteristics of care transfer of adolescents and young adults (AYA).

Transfer characteristics 2023 (four main countries, and total cohort) versus 2019	2023						2019	
	UK	France	Spain	Germany	*p*	Total cohort	Total cohort	*p*
	*n* = 157	*n* = 77	*n* = 67	*n* = 33		*n* = 511	*n* = 1179	
Age of AYA patient transfer								< 0.001^d^
No, see all ages	26.3% (41/156)	16.9% (13/77)	23.1% (15/65)	40.6% (13/32)		26.2% (130/497)	15% (172/1147)	
No, see only adults	5.1% (8/156)	0% (0/77)	0% (0/65)	6.3% (2/32)		5.6% (28/497)	2.3% (26/1147)	
Yes, by 16th birthday	10.3% (16/156)	2.6% (2/77)	24.6% (16/65)	6.3% (2/32)		9.3% (46/497)	11.9% (137/1147)	
Yes, by 17th birthday	7.7% (12/156)	1.3% (1/77)	6.2% (4/65)	3.1% (1/32)		4% (20/497)	4.4% (51/1147)	
Yes, by 18th birthday	8.3% (13/156)	2.6% (2/77)	16.9% (11/65)	21.9% (7/32)		14.7% (73/497)	38.2% (438/1147)	
Yes, by 19th–22nd birthday	4.5% (7/156)	1.3% (1/77)	6.2% (4/65)	0% (0/32)		3.6% (18/497)	2.2% (25/1147)	
Yes, by 23rd–25th birthday	1.9% (3/156)	0% (0/77)	0% (0/65)	0% (0/32)		1.2% (6/497)	1.4% (16/1147)	
Depends	18.6% (29/156)	74% (57/77)	15.4% (10/65)	3.1% (1/32)		23.3% (116/497)	0% (0/1147)	
Other	7.7% (12/156)	0% (0/77)	3.1% (2/65)	3.1% (1/32)		3.8% (19/497)	1.8% (21/1147)	
Evaluation of transition/transfer readiness^a^								
No evaluation tool	20.9% (18/86)	4.8% (3/63)	79.1% (34/43)	41.7% (5/12)	< 0.001^c^	34% (96/282)	41.5% (489/1179)	< 0.001^b^
Parental consent	48.8% (42/86)	84.1% (53/63)	23.3% (10/43)	41.7% (5/12)	< 0.001^c^	44.7% (126/282)	10.3% (122/1179)	< 0.001^b^
Patient consent	40.7% (35/86)	82.5% (52/63)	9% (9/43)	50% (6/12)	< 0.001^c^	46.8% (132/282)	14.5% (171/1179)	< 0.001^b^
Checklist of questions	27.9% (24/86)	84.1% (53/63)	9.3% (4/43)	8.3% (1/12)	< 0.001^c^	33.7% (95/282)	4.2% (50/1179)	< 0.001^b^
Completion transition tool	37.2% (32/86)	77.8% (49/63)	9.3% (4/43)	8.3% (1/12)	< 0.001^c^	34.4% (97/282)	4.1% (48/1179)	< 0.001^b^
Transition committee	10.5% (9/86)	73% (46/63)	4.7% (2/43)	0% (0/12)	< 0.001^c^	22.3% (63/282)		
Feedback system^a^								
No system	36.5% (31/85)	3.2% (2/63)	77.3% (34/44)	54.5% (6/11)	< 0.001^c^	41.1% (116/282)	48.3% (569/1179)	< 0.001^b^
Regular meetings	45.9% (39/85)	85.7% (54/63)	18.2% (8/44)	27.3% (3/11)	< 0.001^c^	43.6% (123/282)	8.6% (101/1179)	< 0.001^b^
Consultation letter back	40% (34/85)	90.5% (57/63)	6.8% (3/44)	18.2% (2/11)	< 0.001^c^	39.7% (112/282)	12.7% (150/1179)	< 0.001^b^
Joint clinics	25.9% (22/85)	88.9% (56/63)	9.1% (4/44)	18.2% (2/11)	< 0.001^c^	34% (96/282)		
Percentage of AYA transferred					0.037^c^			
1%–10%	28.6% (26/91)	6.3% (4/63)	26.1% (12/46)	8.3% (1/12)		22.1% (65/294)		
10%–25%	22% (20/91)	30.2% (19/63)	37% (17/46)	58.3% (7/12)		29.9% (88/294)		
25%–50%	31.9% (29/91)	41.3% (26/63)	21.7% (10/46)	16.7% (2/12)		29.3% (86/294)		
50%–75%	17.6% (16/91)	22.2% (14/63)	8.7% (4/46)	8.3% (1/12)		15% (44/294)		
75%–100%	0% (0/91)	0% (0/63)	6.5% (3/46)	8.3% (1/12)		3.7% (11/294)		
Continuity of care^a^								
Notification of transfer to primary care	45.5% (56/123)	79.2% (57/72)	55.1% (27/49)	34.6% (9/26)	< 0.001^c^	47.8% (192/402)		
Transition report	52.8% (65/123)	77.8% (56/72)	73.5% (36/49)	42.3% (11/26)	< 0.001^c^	58.2% (234/402)		
Clear action plans	48% (59/123)	83.3% (60/72)	53.1% (26/49)	50% (13/26)	< 0.001^c^	55.7% (224/402)		
Comorbidities	44.7% (55/123)	77.8% (56/72)	44.9% (22/49)	50% (13/26)	< 0.001^c^	49.3% (198/402)		
Action plans	47.2% (58/123)	80.6% (58/72)	59.2% (29/49)	57.7% (15/26)	< 0.001^c^	53.2% (214/402)		
Most recent transition assessment	36.6% (45/123)	75% (54/72)	32.7% (16/49)	19.2% (5/26)	< 0.001^c^	35.8% (144/402)		
Direct tel. no. specialist	33.3% (41/123)	73.6% (53/72)	38.8% (19/49)	26.9% (7/26)	< 0.001^c^	38.3% (154/402)		
Red flags	28.5% (35/123)	73.6% (53/72)	46.9% (23/49)	30.8% (8/26)	< 0.001^c^	36.3% (146/402)		
MDT approach	24.4% (30/123)	80.6% (58/72)	20.4% (10/49)	19.2% (5/26)	< 0.001^c^	30.1% (121/402)		
Feedback system	21.1% (26/123)	73.6% (53/72)	24.5% (12/49)	15.4% (4/26)	< 0.001^c^	29.4% (118/402)		
Criteria for AYA transfer^a^								
My clinic refers all patients	4.5% (4/88)	1.6% (1/63)	11.1% (5/45)	8.3% (1/12)	0.005^c^	10.1% (29/287)	13.2% (156/1179)	< 0.001^b^
Any FA	14.8% (13/88)	77.8% (49/63)	24.4% (11/45)	33.3% (4/12)	< 0.001^c^	32.4% (93/287)	7.8% (92/1179)	< 0.001^b^
Multiple FA	30.7% (27/88)	73% (46/63)	44.4% (20/45)	41.7% (5/12)	< 0.001^c^	42.9% (123/287)	15.4% (181/1179)	< 0.001^b^
Multiple FA/asthma	31.8% (28/88)	77.8% (49/63)	55.6% (25/45)	41.7% (5/12)	< 0.001^c^	46.7% (134/287)	19.4% (229/1179)	< 0.001^b^
Multiple FA/asthma/AAI	42% (37/88)	74.6% (47/63)	68.9% (31/45)	66.7% (8/12)	0.005^c^	54.7% (157/287)	22.6% (266/1179)	< 0.001^b^
Anaphylaxis	21.6% (19/88)	68.3% (43/63)	35.6% (16/45)	41.7% (5/12)	< 0.001^c^	37.6% (108/287)	23.4% (276/1179)	< 0.001^b^
FA/AAI	23.9% (21/88)	68.3% (43/63)	48.9% (22/45)	33.3% (4/12)	< 0.001^c^	37.3% (107/287)	16.2% (191/1179)	< 0.001^b^
AIT for resp. allergy	23.9% (21/88)	68.3% (43/63)	60% (27/45)	50% (6/12)	< 0.001^c^	44.6% (128/287)	24.9% (293/1179)	< 0.001^b^
AIT for FA	20.5% (18/88)	69.8% (44/63)	28.9% (13/45)	25% (3/12)	< 0.001^c^	34.8% (100/287)	11.3% (133/1179)	< 0.001^b^
Asthma	14.8% (13/88)	71.4% (45/63)	8.9% (4/45)	0% (0/12)	< 0.001^c^	27.2% (78/287)	10.1% (119/1179)	< 0.001^b^
Difficult/severe asthma	40.9% (36/88)	73% (46/63)	84.4% (38/45)	50% (6/12)	< 0.001^c^	56.1% (161/287)	30.2% (356/1179)	< 0.001^b^
Biologics asthma/CSU/ANG	44.3% (39/88)	81% (51/63)	75.6% (34/45)	41.7% (5/12)	< 0.001^c^	56.4% (162/287)	27.1% (319/1179)	< 0.001^b^
Uncontrolled AD	27.3% (24/88)	74.6% (47/63)	55.6% (25/45)	41.7% (5/12)	< 0.001^c^	44.9% (129/287)	21.6% (255/1179)	< 0.001^b^
AR	9.1% (8/88)	66.7% (42/63)	4.4% (2/45)	8.3% (1/12)	< 0.001^c^	20.2% (58/287)	4.1% (48/1179)	< 0.001^b^
Severe AR	28.4% (25/88)	68.3% (43/63)	68.9% (31/45)	25% (3/12)	< 0.001^c^	42.9% (123/287)	18.5% (218/1179)	< 0.001^b^
Venom allergy	13.6% (12/88)	74.6% (47/63)	53.3% (24/45)	8.3% (1/12)	< 0.001^c^	36.2% (104/287)	16.7% (197/1179)	< 0.001^b^
AIT venom allergy	28.4% (25/88)	74.6% (47/63)	71.1% (32/45)	33.3% (4/12)	< 0.001^c^	47.7% (137/287)	24.1% (284/1179)	< 0.001^b^
EOE						45.3% (130/287)		

*Note:* Countries with more than 30 respondents, UK: *n* = 157, France: *n* = 77, Spain: *n* = 67, Germany: *n* = 33 respondents.

Abbreviations: AAI, adrenaline‐autoinjector; AD, atopic dermatitis; AIT, allergen immunotherapy; ANG, angioedema; AR, allergic rhinitis; CSU, chronic spontaneous urticaria; EOE, eosinophilic oesophagitis; FA, food allergy.

^a^ Participants were allowed to select more than one answer.

^b^ Mann–Whitney *U*.

^c^ Kruskall–Wallis.

^d^ Chi squared.

### 3.3 Improvements Since 2019

Table S1 summarises the key improvements seen when comparing survey results from 2019 to 2023.

In 2023 (compared to 2019) we observed a significant increase in the rate of respondents reporting transition being seen as a priority in their country (44% vs. 19%, *p* < 0.001), having training in transition care (50% vs. 24%, *p* < 0.001), having a transition process (79% vs. 59%, *p* < 0.001)), transition readiness evaluation tools (41.5% vs. 34%, *p* < 0.001) and post‐transfer feedback systems in their practice (48.3% vs. 41.1%, *p* < 0.001). We also observed better rates in 2023 regarding enquiry (‘always asking’) and confidence (‘very confident’) in providing advice about mental health (anxiety, depression, self‐harm), relationships and sexuality issues (20%–27% in 2023 vs. 3%–14% in 2019, *p* < 0.001). Access rates to psychologists, social workers, dietitians and respiratory physiotherapist were significantly higher than those reported in 2019 (28%–55% in 2023 vs. 18%–45% in 2019, *p* < 0.001). Compared to the 2019 survey, a higher number of respondents reported transferring AYA for most clinical criteria/scenarios (*p* < 0.001).

### 3.4 Challenges Faced by HCP

**FIGURE 4:**
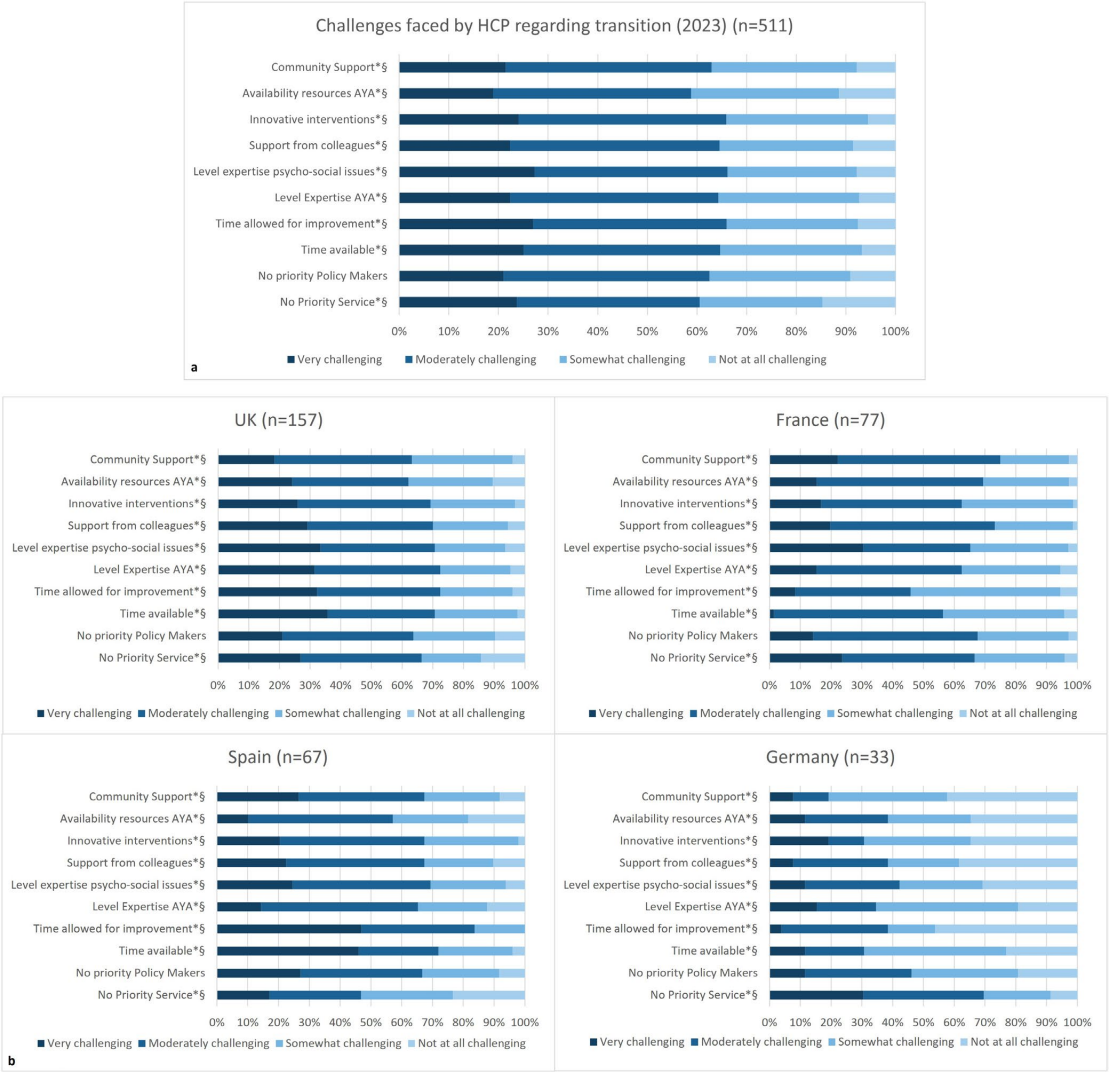
Challenges faced by HCPs when trying to implement an effective transition process for (a) all respondent and (b) for countries with more than 30 respondents. UK: *n* = 157, France: *n* = 77, Spain: *n* = 67, Germany: *n* = 33 respondents. Abbreviations: AYA, adolescents and young adults; HCP, healthcare professionals. 511 participants contributed to the statistical analysis. Symbols: *p value < 0.001; ^§^Kruskall–Wallis.

Over 60% of respondents reported implementing effective transition processes as ‘very’ or ‘moderately challenging’ (Figure 4a). Aspects such as transition not being seen as a priority in their service or by policy makers, the time available in clinic or for service improvements, the support available for innovative interventions or the level of expertise available to identify and address psychosocial issues in AYA were perceived as very challenging by 21%–27% of respondents. Challenges were not assessed in the 2019 survey. The responses to ‘free text’ survey questions did not provide additional information to that already included in the survey questions (data not shown).

### 3.5 Country‐Level Differences

We identified significant differences regarding transition being seen as a priority in the country amongst the main 4 countries (France 88%, UK 49%, Germany 24%, Spain 11%, *p* < 0.001, Table 1). There were also significant differences regarding agreement with the statement ‘transition is important’ (e.g., strongly agree: Spain 67%, Germany 51%, UK 41%, France 21%, *p* < 0.001) and access to training (no training: Spain 82%, Germany 58%, UK 25%, France 18%, *p* < 0.001). In France, 72%–74% respondents reported having completed a dedicated programme, short training and supervision in clinic, whilst this was 8%–25% in Spain and Germany, and 30%–60% in UK (*p* < 0.001). A higher rate of HCP saw patients of ‘all ages’ in their practice in France (78%) compared to Spain, Germany and UK (30%–37%, *p* < 0.001). The rate of HCP with Paediatrics or Paediatric Allergy as background was lower in France (4%–10%) compared to Spain, Germany and UK (24%–57%, *p* < 0.001).

Respondents' familiarity with the guideline and toolbox varied significantly amongst countries (‘not familiar’: Germany 50%, Spain 30%, UK 17% and France 3%, *p* < 0.001, Figure 1b). Regarding adherence to guideline recommendations, availability of transition initiatives and resources for HCPs, significant differences were observed for all recommendations (*p* < 0.001 for all comparisons, Figure 2b). France showed 70%–85% adherence to all recommendations ‘for all patients’, whilst for the vast majority of recommendations this was only 5%–15% in Spain, 25%–35% in Germany and 35%–50% in the UK. Transition guideline, readiness assessment tools, network and/or transition lead were not available for 75%–83% of respondents in Spain compared to 5%–28% in France or UK (*p* < 0.001, see Figure 3b). Significant differences were also observed regarding identifying and addressing psychosocial issues across countries in all areas (*p* < 0.001 for all comparisons, Figure S2b). For example, 68%–78% of respondents from France reported asking ‘always’ or ‘often’ and feeling ‘very confident’ in asking and giving advice on psychological issues, depression, bullying, self‐harm, relationships or sexuality, whilst this was 5%–25% for Spain, 5%–30% for Germany and 10%–30% for the UK (*p* < 0.001). Direct access to allied health/social care professionals (dietitians, psychologists, respiratory physiotherapists, social workers) was higher in France (60–75) compared to Spain (20%–25%), UK (25%–40%) and Germany (25%–50%), *p* < 0.001, Table 1.

Transfer of AYA patients reportedly occurred earlier in Spain (mostly at ages 16–18), whilst in France it was mostly an individualised decision. Most respondents from Spain saw children aged 0–14/0–16/0–18 (57%), whilst in France 78% saw ‘all ages’, Table 2. Reported rates of transfer for the range of given criteria/scenarios was lowest in the UK compared to Spain, France and Germany (*p* < 0.001 for all comparisons). For instance, AYA with difficult/severe asthma, those with multiple food allergies and asthma, and those on biologics for asthma/urticaria are transferred by 42%–44% of respondents from the UK, whilst this is 69%–84% for Spain, 73%–81% for France and 42%–67% for Germany (*p* < 0.001). Strategies to foster continuity of care were more commonly reported by respondents from France compared to the other countries (*p* < 0.001). Having no feedback system following transfer varied from 77% in Spain to 54% in Germany, 36% in UK and 3% in France (*p* < 0.001). Satisfaction with self‐management of AYA care showed no differences between countries (*p* 0.06) (Table 2).

Regarding challenges, we observed differences across countries for most aspects (Figure 4b). For instance, the time available in clinic was perceived as very challenging by 46% of respondents from Spain, 35% from the UK, 12% from Germany and 2% from France. Similarly, the time for service improvements was perceived as very challenging by 46% of respondents from Spain, 32% from the UK, 4% from Germany and 8% from France. Time available in clinic differed amongst countries, with 78% of respondents in Spain having 20 min or less per patient, whilst 85% of respondents from France having more than 20 min (*p* < 0.001).

## 4 Discussion

This survey provides valuable up‐to‐date insight into HCP's perspectives, practice and challenges on transition care following the publication of the EAACI guideline on effective transition in 2020 and the toolbox position paper in 2022. Overall, the survey provides favorable results, with 60%–70% of respondents being familiar with the EAACI guideline and toolbox, and finding them helpful. Around 50% reported these resources had influenced their practice. At least for selected patients, 60%–85% of respondents adhered to the guideline recommendations such as simplifying medication regimes or enrolling families, and 50%–80% had initiatives/resources for transition care such as seeing AYA on their own or producing transition reports.

Compared with the 2019 survey results, the more recent survey provided a more positive picture with almost all HCP's seeing ‘transition as important’ and a priority in their country, with more training and HCPs routinely and confidently asking and advising about some psychosocial aspects. A larger proportion of HCP now transfer AYA to adult care for a range of scenarios, use readiness assessment strategies and feedback mechanisms post‐transfer in 2023 compared to 2019.

Nonetheless, many aspects still show suboptimal results. For instance, 20% of respondents had no transition process, around 50% had no transition‐specific elements in their practice, 40% had no training in transition care or feedback system with adult services and 40%–70% lacked specific components within their service aimed at fostering continuity between specialist services and primary care. Only half of respondents were ‘very’ or ‘moderately satisfied’ with their AYA patients' self‐management. Additionally, 60% found transition care ‘very’ or ‘moderately challenging’, with time constrains and limited expertise on psychosocial issues as leading problems. Also, around half of respondents (only seeing children) reported not transferring AYA with complex disease such as those with severe/difficult asthma, uncontrolled atopic dermatitis, multiple food allergies plus asthma plus adrenaline auto‐injectors, eosinophilic esophagitis or AYA on biologics. This raises concerns about how these patients' needs would be met beyond paediatric services and requires further study and resources.

Importantly, we observed large differences across the main four countries where over 30 responses were obtained. France portrayed the most positive results, including perception of transition as a priority in the country, availability of transition training, familiarity with guidelines and the toolbox, adherence to guideline recommendations, reported practice in identifying and addressing psychosocial issues, direct access to allied HCPs as well as availability of resources such as transition processes, guidelines, readiness assessment tools, post‐transfer feedback mechanisms and regular audit of services. This reflects how the EAACI guidelines could be used in clinical settings. Access to dedicated transition training, for instance in France, might be driving the large differences observed in reported practice across countries. Also, time available for consultations was reported as the longest, and most respondents saw patients of all ages. In contrast, Spain reported largely contrary results with the least time available in clinic. Furthermore, most respondents from Spain had a Paediatrics or Paediatric Allergy background and could only see children up to ages 14 or 16, which is likely to impact service delivery and preparedness prior to transfer. This contrasts with results from France where 78% of HCP coming from a range of backgrounds saw patients of all ages, which might facilitate the transition process. The UK and Germany provided a mixed picture with room for improvement in many areas as above. These findings are supported by a recent global study on distress and access to psychological support for food allergy. The study also found differences across countries, with France having one of the lowest levels of caregivers reporting distress in their child and the UK one of the highest [22]. This underscores the need for harmonization to improve overall care quality [23]. Importantly, any transition recommendations or interventions will need to be tailored to the specific healthcare context. Previous work has demonstrated the importance of considering regional and local factors for effective transition care implementation in rheumatology [24]. A similar approach might be required in the allergy and asthma field.

Access to training on transition care for HCP, particularly regarding psychosocial aspects, is an important area to address. Adolescent medicine and health in general remain inadequately represented as a discipline across Europe, with few dedicated education programmes available. Only three countries offer mandatory training courses in this field for general practitioners [25]. This highlights the need for improved harmonised training in this area. Since the survey was conducted, training modules on the effective transition of AYA with allergies and asthma have been developed by this Taskforce. These are freely accessible on the EAACI website (https://hub.eaaci.org/education/courses/).

HCPs' approach to psychosocial issues in AYA showed a modest improvement in our survey compared to 2019. The significant impact of the COVID‐19 pandemic on young people's mental health [26] might have helped improve awareness and practice in this area. In view of our suboptimal results and concerns arising about young people's mental health (with 1 in 5 people aged 8–25 in the UK having a probable mental health disorder in 2023 [27]).

Evidence clearly shows that successful transition programmes for long‐term conditions translate into better health outcomes [28], including reduced hospital admission rates, decreased need for surgery and improved adult clinic attendance rates [29]. In areas such as type‐I diabetes, juvenile idiopathic arthritis or congenital heart disease, the implementation of transition programmes has led to increased satisfaction with the process and better quality of life [30, 31, 32, 33, 34]. These outcomes are promising as key components of their programmes align with EAACI guidelines on effective transition [35]. However, analysis of transition management of various chronic conditions has revealed significant gaps in practices and reporting standards, similar to our survey findings in allergy in 2019 [15] and now. This emphasises the need for guideline‐compliant approaches and standardised reporting protocols [29, 36]. In response, rheumatologists, for example, developed evidence‐based guidelines and disease‐specific protocols to address these gaps and improve patient outcomes [37]. These insights from other disciplines demonstrate how recognising gaps can drive improvements in transition care across various specialties. Given the ongoing difficulties, limited resources, and competing priorities in healthcare, further work should continue to develop evidence‐based, pragmatic, user‐friendly resources such as allergy and asthma‐specific checklists, readiness assessment tools and educational materials to address AYAs' transition needs in an efficient manner.

The strength of our study is that it repeats the 2019 pan‐European survey and allows the impact of the 2020 EAACI transition guidelines to be assessed. We acknowledge some limitations in our approach. With a survey, there is potential selection bias whereby mainly HCPs with a particular interest in the topic responded, although we would have expected a similar bias in the 2019 survey. Secondly, most respondents were based in tertiary services, and this may not reflect the reality of services elsewhere. Given it was anonymous, we were unable to directly compare individual participant responses in both surveys. Lastly, the number of responses for the four individual countries explored was relatively low, so results should be interpreted with caution.

In conclusion, this survey shows that HCPs' perspectives, training and practice on transition care for AYA with allergies and asthma are modestly improved in comparison to the similar survey prior to the publishing of the EAACI guidelines. For instance, access to specific training increased from 24% in 2019 to 59% in 2023, and transition was reportedly seen as a country priority by 16% of respondents in 2019 and 43% in 2023. However, there are areas that need significant improvement, particularly in certain countries, especially access to training, implementation of evidence‐based guideline recommendations and availability of dedicated resources for improved transition services. Persistent challenges and barriers for implementation also remain.

Society is becoming increasingly aware and worried about the possibility of fatal outcomes in AYA from allergies and asthma, as well as the great burden of living with these conditions. There is a need to equip AYA with the skillset to keep safe, cope well and reach their full potential by providing effective transition. Raising awareness amongst policy‐makers, leadership, colleagues, patients and the community about the need for, and the positive impact of evidence‐based high‐quality transition care remains a key priority. This recognition is likely to influence future policy and funding and, in turn, access to training and resources in practice, in addition to fostering effective collaborative approaches for improved transition care involving all stakeholders.

Further work is required to understand and address the country‐specific barriers and facilitators, promote collaboration and tailor care recommendations to the individual country/regional context. Finally, given the constraints in healthcare resources in many countries, future work should focus on identifying and implementing further evidence‐based cost‐effective pragmatic and sustainable strategies to improve transition care for AYA with asthma and allergies, so that it becomes ‘business as usual’ in daily routines. Developing core training and standards for ‘Adolescent‐friendly’ allergy and asthma health services, adherent with World Health Organisation policies [38] and guideline‐recommendations [17] that services should implement and periodically audit could be a practical step to drive improvements in transition care. This Taskforce is committed to contributing to these multifaceted initiatives with the support of the EAACI.

## Author Contributions

Study concept and design: G.R., M.V.O., N.A. Statistical analysis and interpretation of data: M.V.O., N.A. Reviewed and edited the manuscript: G.R., M.V.O., E.K., E.A., K.B., R.C.K., P.C., C.A., B.D., C.G.M., A.D., C.G., B.E., H.P., A.F.S., M.P., T.G.‐B., S.S.G., M.H.G., O.P. All authors provided critical review of the manuscript. All authors read and approved the final manuscript.

## Ethics Statement

The study was approved by the Ethics and Research Governance Committee (ICREC Reference number: 6489853) at Imperial College London, United Kingdom.

## Conflicts of Interest

The authors declare no conflicts of interest.

## Supporting information


**Table S1.** Main Improvements identified between 2019 and 2023. Abbreviations: AAI, adrenaline autoinjector; AD, atopic dermatitis; AIT, allergen immunotherapy; ANG, angioedema; AR, allergic rhinitis; CSU, chronic spontaneous urticaria; FA, food allergy; Resp., respiratory. ^†^Chi‐squared; ^‡^Mann–Whitney *U*.
**Figure S1.** Study flowchart of sample selection. Abbreviations: EAACI, European Academy of Allergy and Clinical Immunology; NAS, National Allergy Societies.
**Figure S2.** Healthcare professionals’ practice on psychosocial aspects when managing AYA with allergy and asthma across Europe. (a) Results of the 2023 survey; (b) Comparison of the 2023 survey results amongst the countries with over 30 responses (UK, Spain, France, Germany); Results of the 2019 survey and comparison with the 2023 survey. Abbreviations: Psychol/Issues, psychological issues; QoL, quality of life. Symbols: *p value < 0.001; ^§^Kruskall–Wallis; ^†^Chi‐squared test.

## Data Availability

The datasets used and/or analyzed during the current study are available from the corresponding author on reasonable request.
