# Supplementary material for: Improved Transition Management of Adolescents and Young Adults With Allergy and/or Asthma: An EAACI Task Force Report on a Follow‐Up European Survey
Source: Allergy. 2025 Jun 17;80(6):1592–612. doi: 10.1111/all.16603 (PMC12186585; doi:10.1111/all.16603)
Supplement: Supplementary file 1 — Table S1. Main Improvements identified between 2019 and 2023. Abbreviations: AAI, adrenaline autoinjector; AD, atopic dermatitis; AIT, allergen immunotherapy; ANG, angioedema; AR, allergic rhinitis; CSU, chronic spontaneous urticaria; FA, food allergy; Resp., respiratory. †Chi‐squared; ‡Mann–Whitney U. Figure S1. Study flowchart of sample selection. Abbreviations: EAACI, European Academy of Allergy and Clinical Immunology; NAS, National Allergy Societies. Figure S2. Healthcare professionals’ practice on psychosocial aspects when managing AYA with allergy and asthma across Europe. (a) Results of the 2023 survey; (b) Comparison of the 2023 survey results amongst the countries with over 30 responses (UK, Spain, France, Germany); Results of the 2019 survey and comparison with the 2023 survey. Abbreviations: Psychol/Issues, psychological issues; QoL, quality of life. Symbols: *p value < 0.001; §Kruskall–Wallis; †Chi‐squared test. [file ALL-80-1592-s001.docx]

**Additional file**

**Contents**

Table S1: Improvement Highlights 2023 vs 2019 3

Figure S1: Flow Chart 5

Figure S2: Psychosocial aspects 7

Questionnaire 11

Table S1. Main Improvements identified between 2019 and 2023.

Abbreviations: FA, Food Allergy, AAI, Adrenaline Autoinjector, Resp., Respiratory, CSU, Chronic spontaneous urticaria, ANG, Angioedema, AD, Atopic Dermatitis, AR, Allergic Rhinitis, AIT, Allergen immunotherapy. † Chi square, ‡ U-Mann-Whitney.

Figure S1: Study flowchart of sample selection.


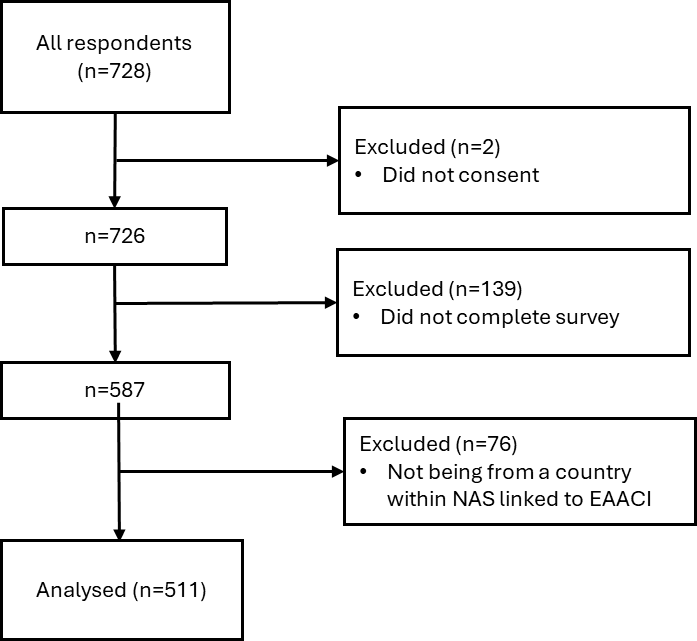


Abbreviations: EAACI: European Academy of Allergy and Clinical Immunology, NAS: National Allergy Societies

**Figure S2.** Healthcare professionals’ practice on psychosocial aspects when managing AYA with allergy and asthma across Europe. (a) Results of the 2023 survey; (b) Comparison of the 2023 survey results among the countries with over 30 responses (UK, Spain, France, Germany); Results of the 2019 survey and comparison with the 2023 survey.


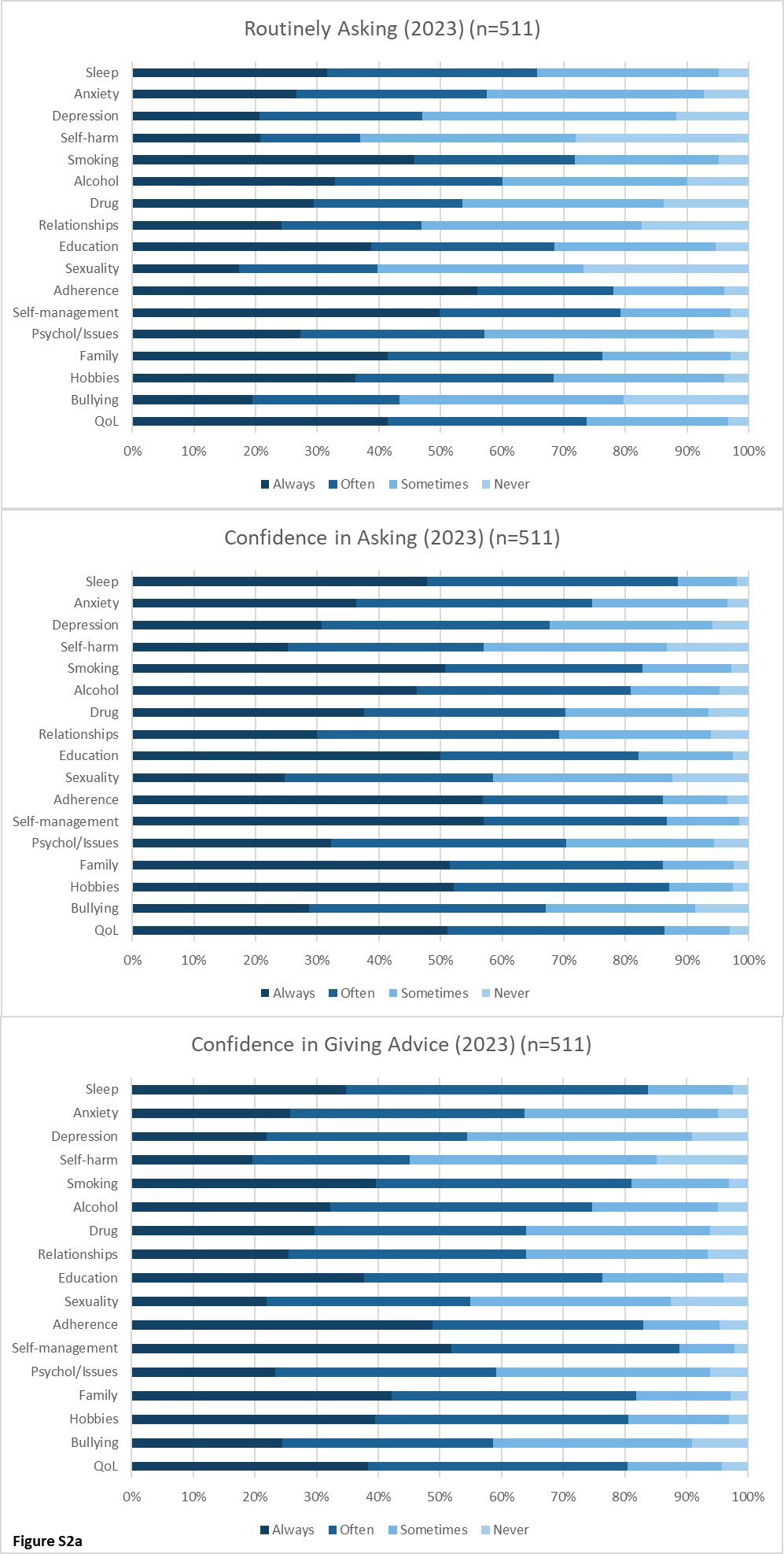


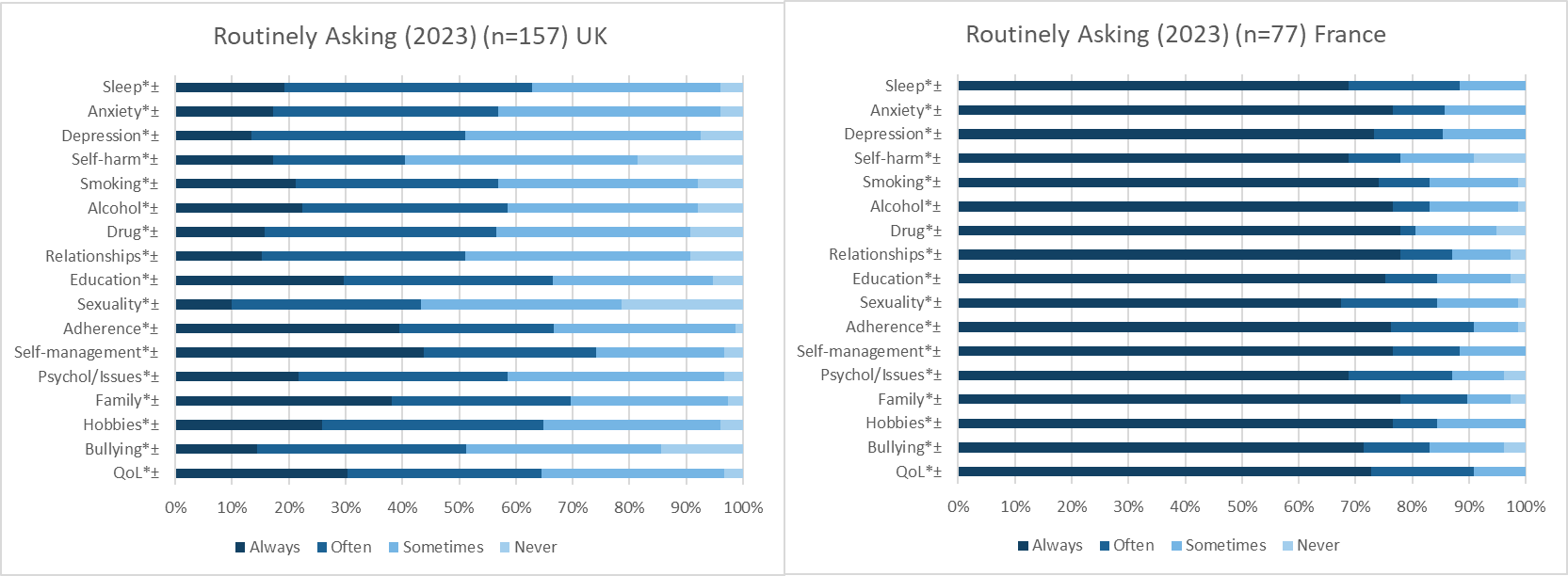


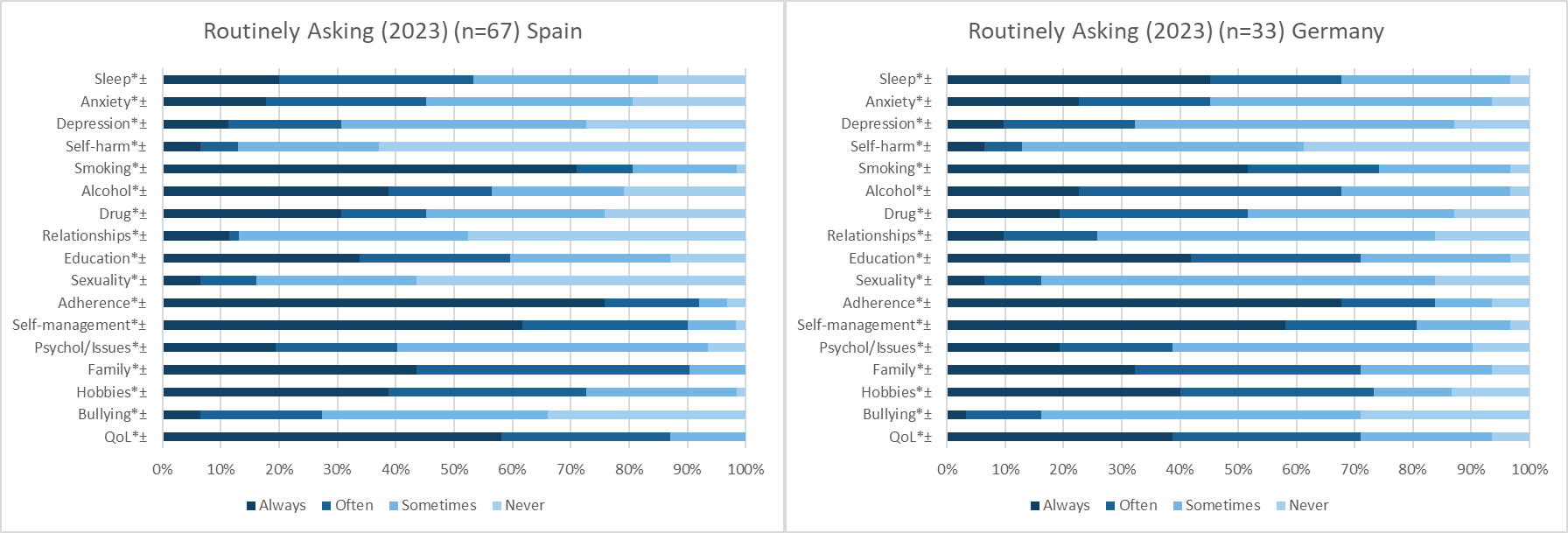


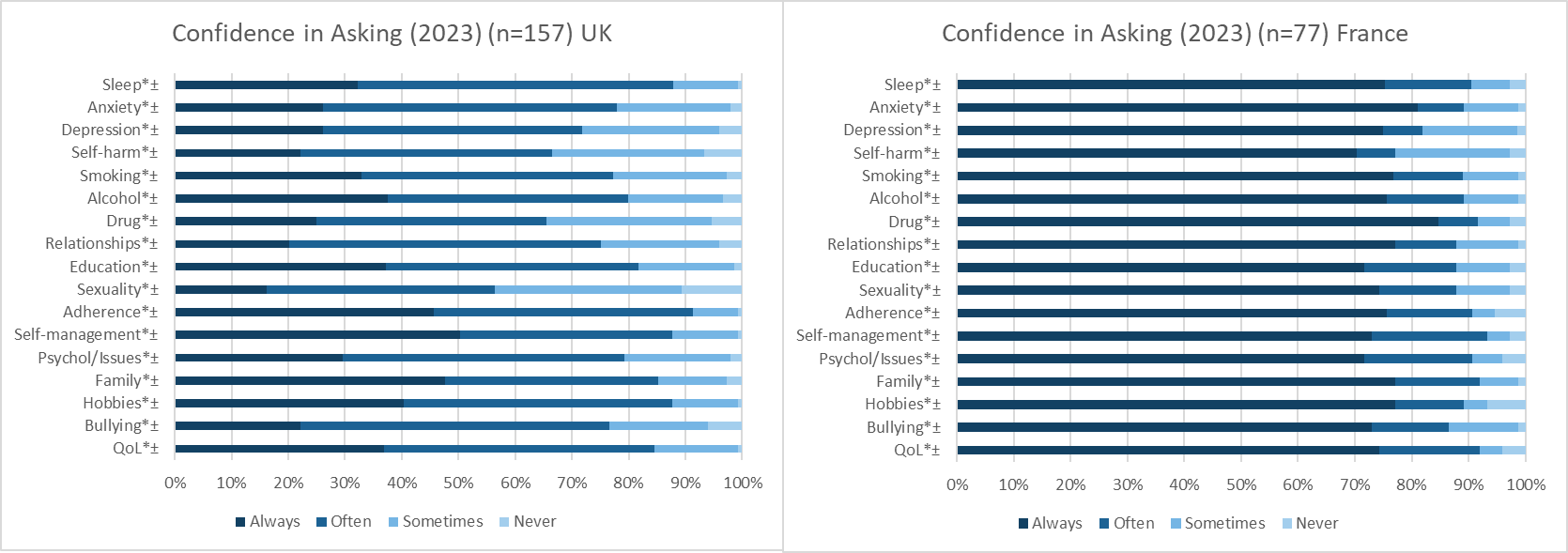


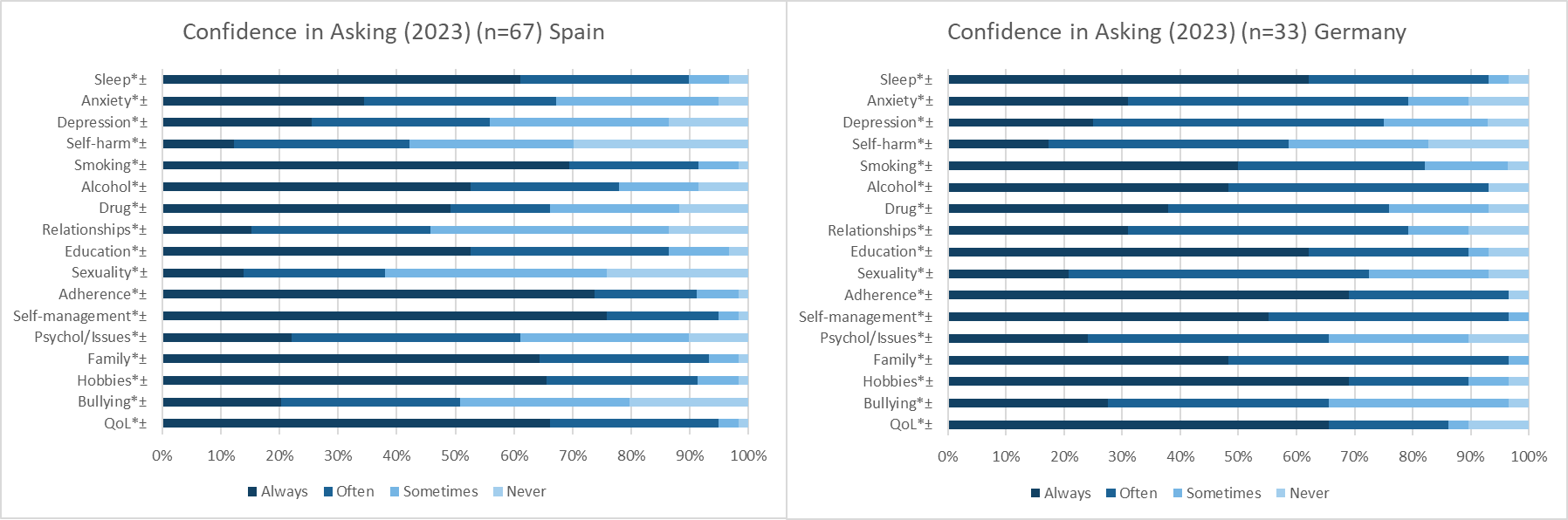


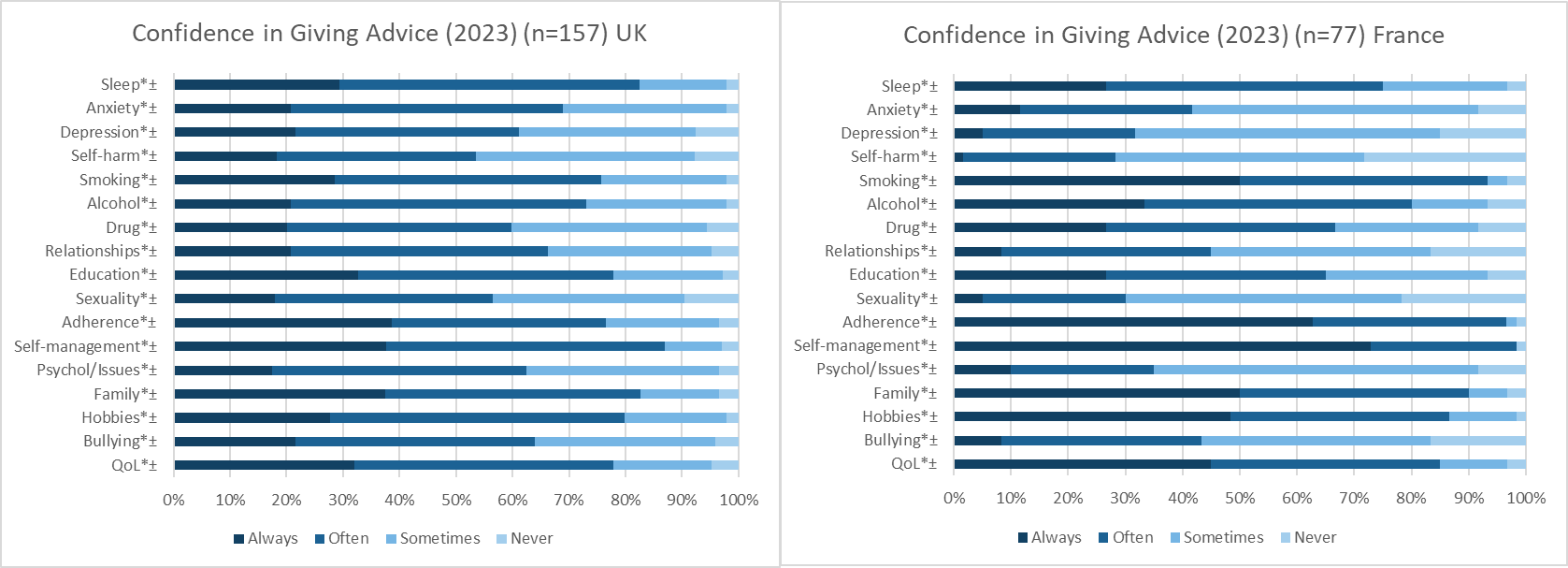


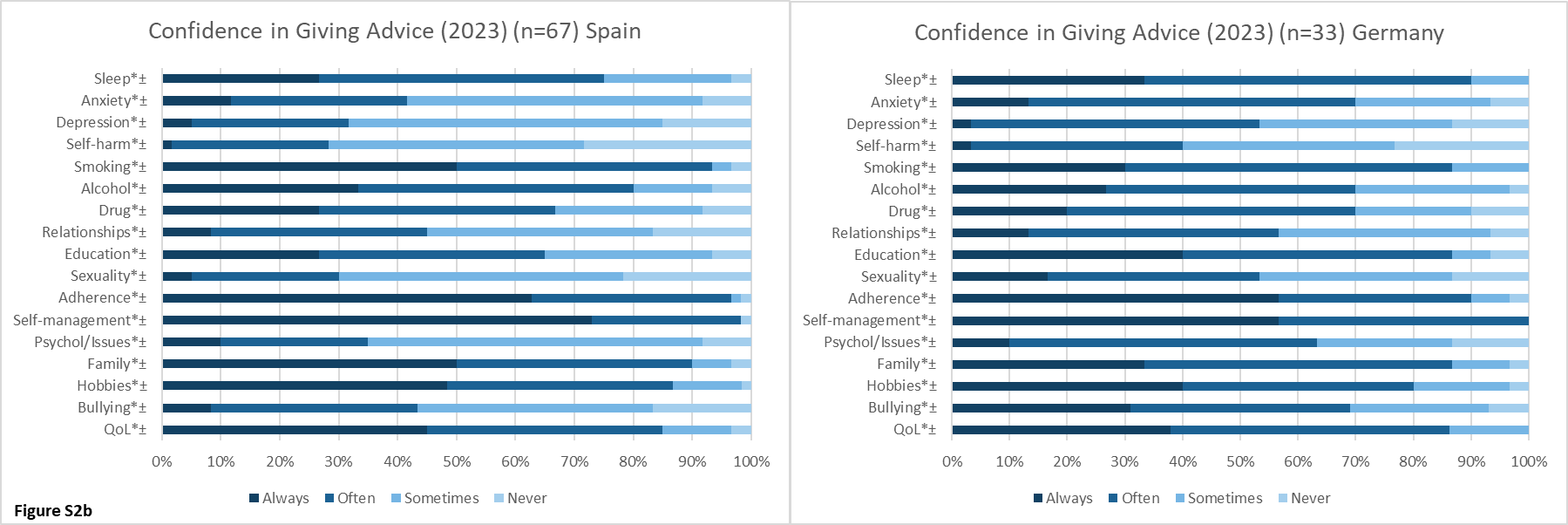


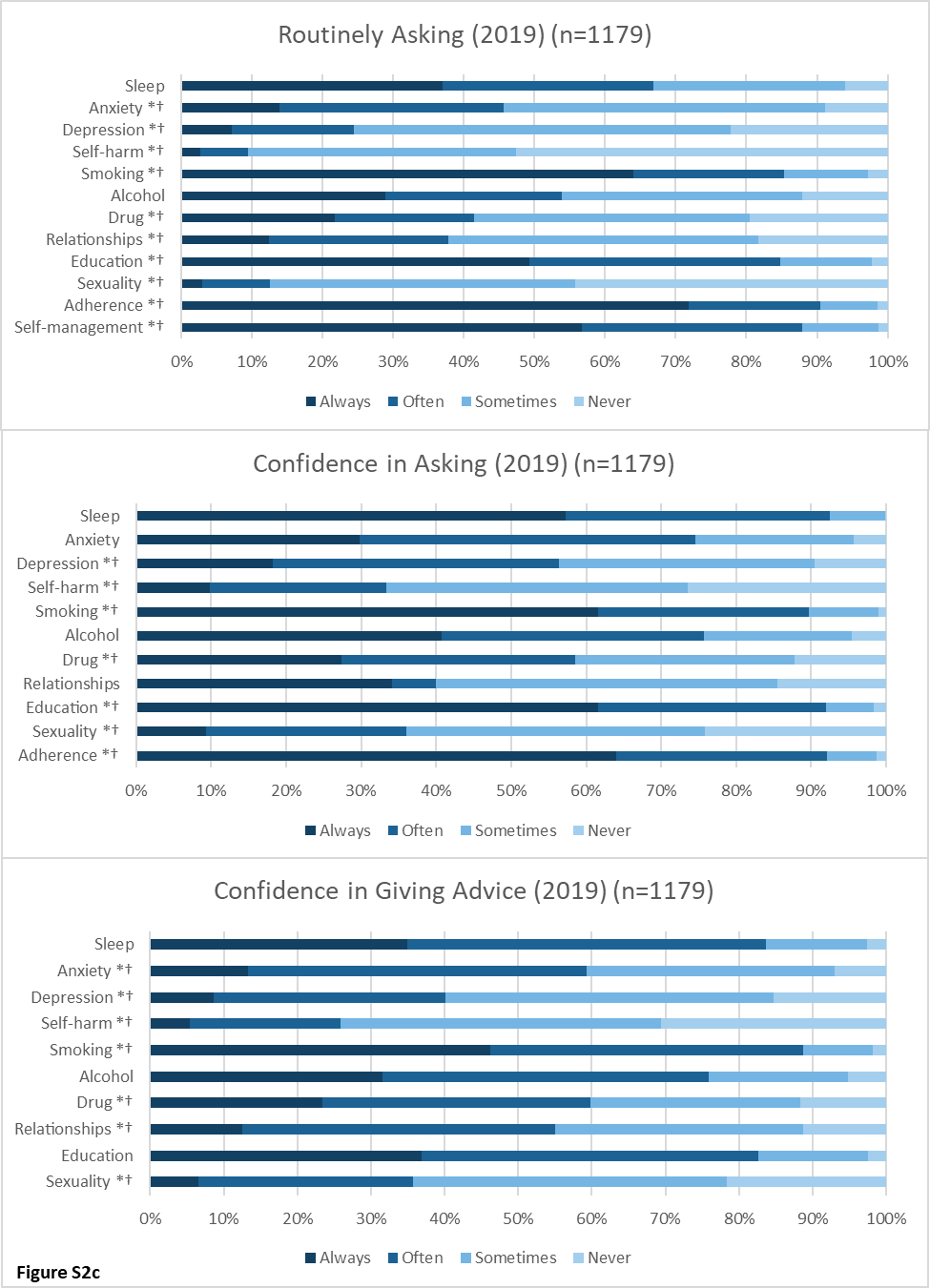


Abbreviations: Psychol/Issues: Psychological issues, QoL: Quality of life. Symbols: * p value <0.001;§ Kruskall-Wallis; † Chi square test.

**Original survey in full**

**Survey title:** Lost in transition?

You are being invited to take part in a survey as part of a research study. Before you decide it is important for you to understand why the research is being done and what it will involve. Please take time to read the following information carefully and discuss it with others if you wish.

Adolescents and Young Adults (AYA) with allergies and asthma may have challenges that impact on self-management and their needs are different to those of other age groups. Adolescence is an important phase to promote healthy behaviour, to prevent health risks and empower AYA to become competent adult patients. In the last years, the European Allergy and Clinical Immunology (EAACI) Taskforce on AYA has published a guideline on the effective transition of AYA with allergies and/or asthma, and a Toolbox paper to help implement this into clinical practice. We would like to invite you to complete a short survey (10 minutes) to allow us to understand your perspectives and current practice on AYA care.

An open access publication of the results is planned and will be supported by EAACI. Please note, no personally identifiable information will be included with responses (i.e., IP address, email addresses). Respondents cannot be identified. Thanks so much for taking the time to complete the survey.

1. I confirm that I have read and understand the Intro paragraph of the Survey with the Title: Lost in Transition? and have had the opportunity to ask questions which have been answered fully

2. I understand that my participation is voluntary, and I am free to withdraw at any time, without giving any reason and without my legal rights nor treatment / healthcare being affected.

3. I consent to take part in the Survey with the Title: Lost in Transition? (Required question)

Yes, I agree.

No, I do not agree

Part 1. Demographics and clinical setting

1.1. Which EAACI Section/Interest Group are you affiliated with? (tick just one)

• Asthma

• Dermatology

• ENT

• Immunology

• Paediatrics

• Primary Care and Allied Health

• None

1.2. In which country do you work?

• Austria

• Belarus

• Belgium

• Bulgaria

• Croatia

• Cyprus

• Czech Republic

• Denmark

• Estonia

• Finland

• France

• Germany

• Greece

• Ireland

• Italy

• Latvia

• Lithuania

• Luxembourg

• Malta

• Netherlands

• Norway

• Poland

• Portugal

• Russia

• Romania

• Slovakia

• Slovenia

• Spain

• Sweden

• Switzerland

• United Kingdom

• Ukraine

• Türkiye

• Other, please name (free text)

1.3. What is your profession?

• Doctor

• Specialist allergy nurse

• Dietitian

• Psychologist

• Other, please state (free text)

1.4. Please select your specialty (tick all that apply)

• Paediatric allergy

• Paediatrics

• Allergy (adults only)

• Allergy (children and adults)

• Dermatology

• Respiratory Medicine

• ENT

• General Practitioner

• Gastroenterologist

• Other, please specify (free text)

1.5. In which setting do you work? (tick all that apply)

• Tertiary care (e.g., highly specialised teaching hospital)

• Secondary care (e.g., hospital setting with some degree of specialisation)

• Primary care (e.g., outpatient clinic, walk-in clinic)

• Private practice

• Other, please describe (free text)

1.6. What age range does your service cover?

• All ages

• 0-14

• 0-16

• 0-18

• 12 or over

• 14 or over

• 16 or over

• 18 or over

• Other, please specify (free text)

1.7. How much time do you have for your usual follow-up consultation with AYA?

• Up to 10 min

• Up to 20min

• Up to 30min

• Up to 45min

• Over 45min

1.8. Does your service have direct access (that is, without being referred by a doctor or other healthcare practitioner) to the following healthcare professionals? (tick all that apply)

• Allergy / asthma nurse

• Dietician

• Paediatric allergist

• Adult allergist

• Psychologist

• Respiratory physiotherapist

• Social worker

• Gastroenterologist

• Pulmonologist

• Dermatologists

• Audiologist

• Ophthalmologist

• Others, please specify (free text)

Part 2. Perspectives and professional development on transition care

2.1. To what extent do you agree with the statement, transition is important for AYA with allergies or asthma. (tick as appropriate)

• Strongly agree

• Agree

• Neither agree nor disagree

• Disagree

• Strongly disagree

2.2. Does the department of health in your country make transition a high priority?

• Yes

• No

• Don’t know

2.3. Have you had any specific training in the care of AYA? (tick all that apply)

• Dedicated training programme on Adolescent Medicine

• Short training/education course

• Supervision within your clinical role

• No specific training

• Other, please specify (free text)

2.4. EAACI published a guideline on the effective transition of AYA with allergy and asthma in 2020.

How familiar are you with the content of the guideline?

1= very familiar

2= moderately familiar

3= somewhat familiar

4= not familiar at all

2.5. How useful did you find the guideline in your practice?

1= very useful

2= moderately useful

3= somewhat useful

4= not useful at all

2.6. To what extent has the guideline influenced your approach to transition in practice?

1= very much influenced my approach

2= moderately influenced my approach

3= somewhat influenced my approach

4= did not influence my approach at all

2.7. EAACI published a Toolbox position paper on useful resources to support the effective transition of AYA with allergy and asthma in 2022.

How familiar are you with the content of the Toolbox?

1= very familiar

2= moderately familiar

3= somewhat familiar

4= not familiar at all

2.8. How useful did you find the Toolbox in your practice?

1= very useful

2= moderately useful

3= somewhat useful

4= not useful at all

2.9. To what extent has the Toolbox influenced your approach to transition in practice?

1= very much influenced my approach

2= moderately influenced my approach

3= somewhat influenced my approach

4= did not influence my approach at all

Part 3. Transfer

3.1. Are AYA transferred to adult services from your service, and if so, by what age?

• No, my clinic does not transfer patients into adult services because we see all ages.

• No, my clinic does not transfer patients into adult services although we only see children

• No, my clinic sees only adult patients

• Yes, by their 16th birthday

• Yes, by their 17th birthday

• Yes, by their 18th birthday

• Yes, by their 19th-22nd birthday

• Yes, by their 23rd -25th birthday

• It depends on each patient

• Other, please specify (free text)

3.2. Please estimate what percentage of your patients are transferred to specialist adult services for their allergy or asthma care:

• 1-10%

• 10-25%

• 25-50%

• 50-75%

• 75-100%

3.3. Do you know how many of your transition patients regularly attend the adult clinic after referral:

• Yes, please specify the percentage (free text)

• No

3.4. Which criteria should AYA meet in your department to be transferred to specialist adult services rather than returned to primary care? (tick all that apply)

• Not applicable, my clinic refers all patients to adult services

• All paediatric patients with any food allergy

• All paediatric patients with multiple food allergies

• All paediatric patients with multiple food allergies and asthma

• All paediatric patients with multiple food allergies, asthma and adrenaline autoinjector

• All paediatric patients who have ever experienced anaphylaxis

• All paediatric patients with food allergy who also have an adrenaline auto-injector

• All paediatric patients on immunotherapy for respiratory allergy

• All paediatric patients who have gone through immunotherapy for food allergy

• All paediatric patients with asthma

• All paediatric patients with difficult, severe, or poorly controlled asthma

• All paediatric patients on biologics for asthma or chronic spontaneous urticaria/angioedema

• All paediatric patients with severe or uncontrolled atopic dermatitis

• All paediatric patients with allergic rhinoconjunctivitis

• All paediatric patients with severe allergic rhinoconjunctivitis

• All paediatric patients with hymenoptera venom allergy

• All paediatric patients on immunotherapy for hymenoptera venom allergy

• All paediatric patients with Eosinophilic Esophagitis

• Other, please specify (free text)

3.5. How do you evaluate whether a patient is ready to be sent to adult services? (tick all that apply)

• We have no evaluation tool; patients are transferred at a specific age

• Parental consent

• Patient consent

• Checklist of questions/knowledge that the patient completes

• Completion of ‘ready, steady, go’ or similar adolescent transition tool

• Transition Committee consent

• Other, please specify (free text)

3.6. Is there a feedback system between your paediatric service and your local adult service? (tick all that apply)

• No system of feedback in place

• Regular meetings to discuss patients

• The consultation letter from the first visit to the adult clinic is sent back to the referring paediatrician

• Joint clinics or visits at the same time (patient, parents, adult and paediatric doctors and nurses specialized in the transition process)

Part 4. Psychosocial aspects

4.1. When you see AYA in your practice, do you routinely ask about the following areas (tick as appropriate)

Always Often Sometimes Never

Sleep

Anxiety

Depression

Self-harm

Psychological issues in general

Smoking

Alcohol use

Drug use

Relationships

Education/Employment

Sexuality

Adherence

Confidence in managing their own allergies and/or asthma

Family/Caregivers

Hobbies

Bullying

Quality of Life

4.2. When you see AYA in your practice, how confident do you feel to ask about the following areas: (tick as appropriate)

Very confident Quite confident Not very confident Not confident

Sleep

Anxiety

Depression

Self-harm

Psychological issues in general

Smoking

Alcohol use

Drug use

Relationships

Education/Employment

Sexuality

Adherence

Confidence in managing their own allergies and/or asthma

Family/Caregivers

Hobbies

Bullying

Quality of Life

4.3. When you see AYA in your practice, how confident do you feel that you can provide good/relevant advice for the following areas: (tick as appropriate)

Very confident Quite confident Not very confident Not confident

Sleep

Anxiety

Depression

Self-harm

Psychological issues in general

Smoking

Alcohol use

Drug use

Relationships

Education/Employment

Sexuality

Adherence

Confidence in managing their own allergies and/or asthma

Family/Caregivers

Hobbies

Bullying

Quality of Life

Part 5. Approach and resources for transition

5.1. At what age do you usually start the transition process (i.e., patient preparation for self-management in adulthood plus/minus preparation for adult healthcare services)?

• My clinic or service doesn’t have a transition process

• 10-12 years

• 12-14 years

• 14-16 years

• 16-18 years

• It depends on each patient

• Other, please specify (free text)

5.2. How satisfied are you generally about the quality of AYA’s self-management of their allergies and asthma in your practice (tick as appropriate)

• Very satisfied

• Moderately satisfied

• Somewhat satisfied

• Not at all satisfied

• Not sure

5.3. In your service, which of the components listed are available specifically for AYA with allergies and asthma? (tick all that apply)

Yes, for all patients Yes, for selected patients only Not available

• Transition guideline/specific protocol for healthcare professionals

• Transition readiness assessment tool (e.g. Ready Steady Go, Transition Readiness Assessment Questionnaire, ADAPT Survey, Leaving Home)

• Educational Workshops

• Peer learning/peer support for patients

• Webinars

• Selected web-based information resources or e-Learning materials

• Phone hotline

• Consultation without parents present (e.g., at certain age parents are asked to leave the consultation room and the patient seen independently)

• Joint transition clinics with the paediatric and adult services

• Transition lead

• Transition network

• Regular meetings involving paediatric and adult services in the field of allergy and pneumology to discuss AYA patients

• Transition report

• Consultation letters or reports are sent to paediatric or adult colleagues involved in individual patients’ care

• Communication (post, emails, texts) addressed directly to AYA (e.g., medical reports, letters, appointments)

• Strategies to address the psychological impact of AYA such as Motivational Interviewing or Cognitive Behavioural Therapy

• We have no specific resources/elements for AYA

• Other, please tell us (free text)

5.4. In your service, which of the components listed are you using in your clinical practice? (tick all that apply)

Yes, for all patients Yes, for selected patients only Not available

• Starting transition process from an early and developmentally appropriate age (usually age 11-13)

• Structured multidisciplinary team approach

• Routinely seeing AYA by themselves at least for part of the appointment

• Personal action plans

• Simplifying medication regimes

• Active monitoring of adherence (e.g., smart inhalers, medication reminders, mobile applications)

• Psychosocial assessment tools (e.g., HEEADSSS)

Patient reported outcome measures (e.g., health related quality of life questionnaires)

• Signposting to high quality online resources (e.g., good patients’ organisations)

• Enrolling the family in assisting AYA to take on their self-management

• Encouraging AYA to let their friends know about their disease

• Encouraging families to change their routines to support AYA

• Promoting allergy/asthma awareness among peers/co-workers/teachers/managers

• Promoting allergy/asthma support groups/interest groups for patients, parents, and carers

• Does your service offer specific training in transitional and AYA care for healthcare professionals?

• Does your service offer regular audit of transition service?

• Other, please tell us

5.5. Which of the components listed do you have in your service to foster continuity of care between specialised services and primary/community care during the transition process? (tick all that apply)

• Notification of AYA being transferred to adult services or primary care

• Transition report including medical summary to primary care providers

• Clear actions on prescribing

• Clear actions on comorbidities needing optimisation

• Relevant Emergency/ Actions plans distributed to primary care providers

• Most recent transition readiness assessment distributed to primary care providers

• Direct access telephone number to contact specialist

• Red flags for primary care providers when specialist should be recontacted

• An established multidisciplinary team with input from paediatric, adult, and primary care providers

• Feedback system from adult and/ or paediatric services to primary care about AYA`s attendance, circumstances, and major changes in management

• Other, please tell us (free text)

5.6. There may be challenges when a service is trying to implement an effective transition process. In your practice, how challenging are each of the factors listed?

1= very challenging

2= moderately challenging

3= somewhat challenging

4= not at all challenging

• Transition not seen as a need in my service as we see children and adults

- Transition not seen as a priority by policy makers and/or colleagues

• Time available in clinic

• Time allowed to develop or improve existing services

• Level of expertise on AYA particularities, challenges and needs

• Level of expertise to identify and address psycho-social issues in AYA

• Support from colleagues to establish a multidisciplinary transition network or pathway

• Level of expertise or support to establish innovative interventions such as peer-led interventions

• Availability of practical resources for AYA with asthma and allergies in your own language (e.g., advice sheets, action plans)

• Level of support from the community (school staff, sport club staff) to help with AYA transition

5.7. Please let us know about any other thoughts you have about the transition process.

(free text)

Closing paragraph which is linked to this survey in a separate survey and includes the following wording:

Thank you for completing the survey.

If you want to participate in an Amazon Voucher Prize Draw, please leave your email here.

Please note, your responses will not be linked to your email address, data will be deleted after the draw and prior to analysis in order to maintain anonymity.

Nina Atzert, Marta Vazquez-Ortiz and Graham Roberts on behalf of the EAACI Adolescent and Young Adult taskforce
